# In vitro models for testicular steroidogenesis: current status and future perspectives

**DOI:** 10.1007/s00204-026-04380-5

**Published:** 2026-04-28

**Authors:** Eliška Řehůřková, Lola Bajard, Iva Sovadinová

**Affiliations:** Faculty of Science, RECETOX, Masaryk University, Kotlarska 2, 611 37 Brno, Czech Republic

**Keywords:** Alternative testing strategies, Chemical safety assessment, Endocrine disruptors, Male fertility, Male reprotoxicants, New approach methodologies

## Abstract

Testicular steroidogenesis is fundamental to male reproductive health, but its disruption by environmental and emerging chemicals remains insufficiently characterized due to limitations in existing test systems. Traditional animal models pose ethical and logistical challenges, while the validated H295R assay—based on a female adrenal carcinoma cell line—fails to reflect male gonadal steroidogenesis. This review uses a semi-systematic approach to evaluate over 1500 studies employing in vitro models, including primary Leydig cells, Leydig cell lines, stem cell-derived Leydig-like cells, and advanced 3D testicular systems. We assess species origin, developmental relevance, culture conditions, and the extent to which these models replicate key steroidogenic pathways. Most models rely on rodent-derived, cancerous cell lines cultured in two-dimensional monolayers, with limited representation of human and immature Leydig cells. A targeted full-text analysis examined the effects of 23 reference chemicals on testosterone, progesterone, androstenedione, and estrogen levels across the H295R assay and eight testicular in vitro models. Forskolin, genistein, prochloraz, and ketoconazole showed consistent effects and may serve as promising reference compounds. However, data for most chemicals in testicular models are scarce or inconsistent—particularly for androstenedione and progesterone—underscoring the need for improved model standardization. We propose future directions to enhance predictive power, including the development of hormone-responsive, species- and stage-specific models cultured under hormone-controlled conditions. Such advances are essential to improve chemical safety assessment and facilitate regulatory acceptance of alternative test methods.

**Supplementary Information:**

The online version contains supplementary material available at 10.1007/s00204-026-04380-5.

## Introduction

Reproductive health is essential for individual well-being and population sustainability, yet evidence of its decline is mounting. Infertility affects 8–12% of couples worldwide (Kumar and Singh 2015). Gonadal steroid hormones regulate reproductive function, fertility, neurodevelopment, physical traits, and behavior, and their levels are tightly controlled during critical developmental windows (Ernie 2005). This hormonal balance is vulnerable to disruption by environmental and emerging chemicals, collectively known as endocrine-disrupting compounds (EDCs) (Thomas Zoeller et al. 2012; WHO and UNEP, 2012).

Among the most affected targets are Leydig cells, which produce over 90% of androgens in males (Winters 2020). Disruption of Leydig cell function can lead to urogenital abnormalities, erectile dysfunction, reduced sperm quality and quantity, subfertility or infertility, and even testicular cancer (Skakkebaek et al. 2001; Yeo et al. 2021). The prevalence of hypogonadism, characterized by diminished testosterone production and testicular function, is rising (Yeo et al. 2021).

**Fig. 1:**
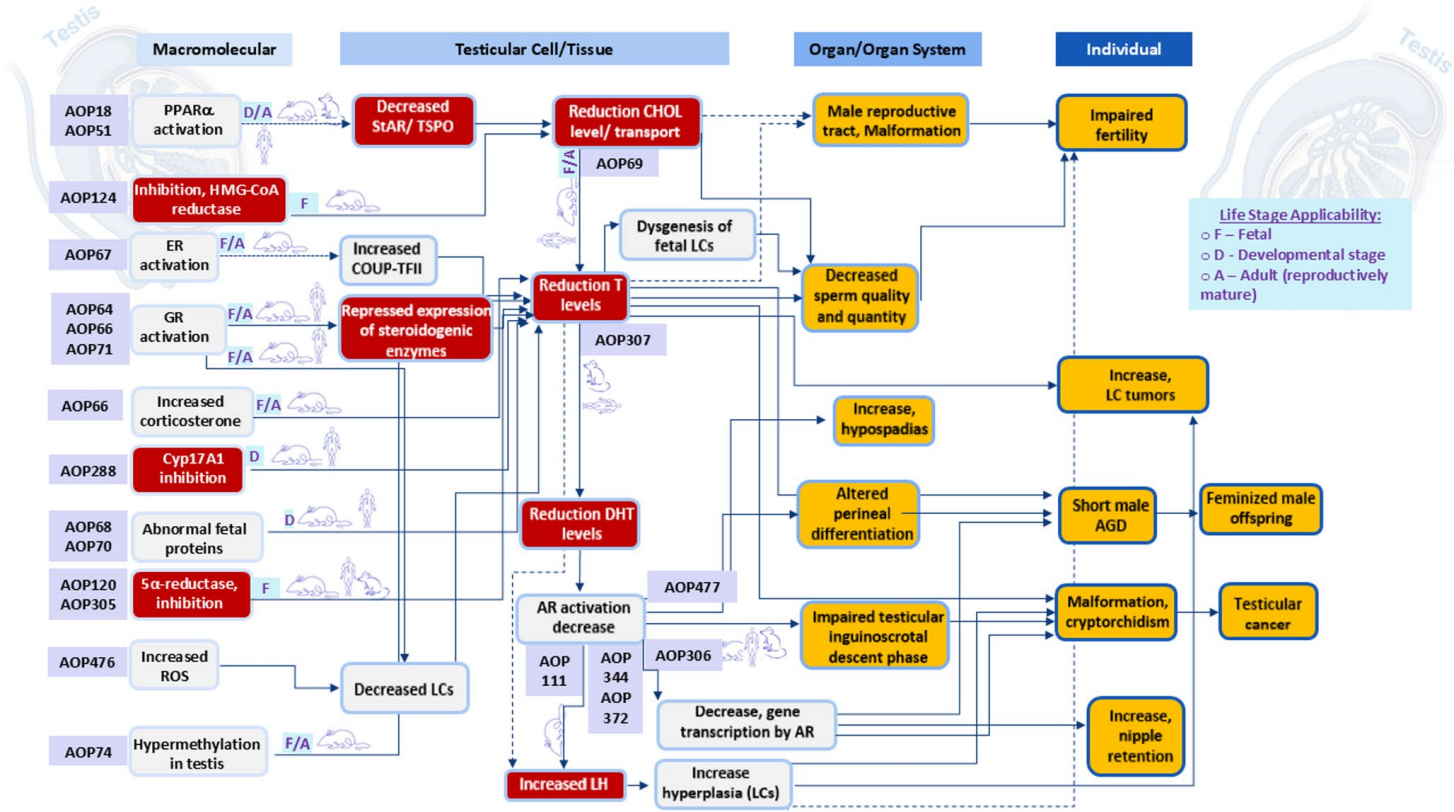
Simplified adverse outcome pathway (AOP) network for testicular steroidogenesis, highlighting Leydig cell key events (KEs) and their corresponding adverse outcomes (AOs). All information, including applicability domains, is sourced from the AOP wiki (https://aopwiki.org/). KEs specific to male steroidogenesis, such as cholesterol transport, StAR (steroidogenic acute regulatory protein) and TSPO (translocator protein) expression, and initial enzymatic steps (e.g., CYP11A1 activity), are highlighted in red. Solid lines indicate direct, adjacent relationships between events; dashed lines represent non-adjacent connections. Most AOPs are preliminary and under development, with only AOP18 currently under formal review.

Altered testicular steroidogenesis and Leydig cell hormone output are key events within the Adverse Outcome Pathway (AOP) framework (Ankley et al. 2010) (Fig. 1). The AOP-Wiki lists 22 related AOPs, most still under development, addressing disruptions such as inhibition of HMG-CoA (3-hydroxy-3-methylglutaryl coenzyme-A) reductase, downregulation of StAR (steroidogenic acute regulatory protein) and TSPO (translocator protein), impaired LH (luteinizing hormone) signaling, disrupted cholesterol transport, and reduced activity of steroidogenic enzymes (CYP11A1, CYP17A1, 3β-HSD, 17β-HSD3). These changes can ultimately reduce testosterone and dihydrotestosterone synthesis, leading to adverse outcomes including hypospadias, cryptorchidism, infertility, nipple retention, and testicular tumors. Recently, Bouftas et al. (2026) introduced an upstream AOP network for disrupted steroidogenesis, emphasizing reduced enzyme activity and hormone production. Integrating Leydig cell-specific in vitro models into such networks could enhance mechanistic interpretation and regulatory relevance. Our review complements this effort by evaluating biological models needed to populate these networks, particularly for male-specific pathways and developmental stages underrepresented in current AOP-Wiki content.

Studying the impact of chemicals on male reproductive health is constrained by the limited availability of physiologically relevant models. Current regulatory assessments rely on validated assays with established relevance and reliability (OECD 2005). Most validated methods for identifying male reproductive toxicants and EDCs depend on animal testing, as outlined in OECD Test Guidelines (TG) 421, 416, and 443, and Guidance Document (GD) 150 (OECD 2018a, b, c, d). However, traditional animal tests are costly, time-consuming, ethically contentious, and lack high-throughput capacity and direct human relevance (Krewski et al. 2010). These limitations underscore the urgent need for animal-free alternative approaches–a shift increasingly supported by toxicologists and regulatory agencies.

For assessing androgen pathway effects, OECD GD150 lists validated in vitro methods such as androgen receptor (AR) (anti)agonist assays and the steroidogenesis assay (OECD 2018d). However, Kleinstreuer et al. (2018) demonstrated that AR (anti)agonist screening alone does not fully capture chemicals identified as antiandrogenic in the in vivo Hershberger assay. Discrepancies in LOEL (Lowest Observed Effect Level), IC_50_ (Half Maximal Inhibitory Concentration), and metabolic capacity may explain some discrepancies, but alternative modes of action affecting androgen levels are also likely to contribute. This highlights the importance of incorporating hormone level measurements in in vitro test batteries to identify anti-androgenic chemicals and potential male reproductive toxicants for regulatory purposes.

**Fig. 2:**
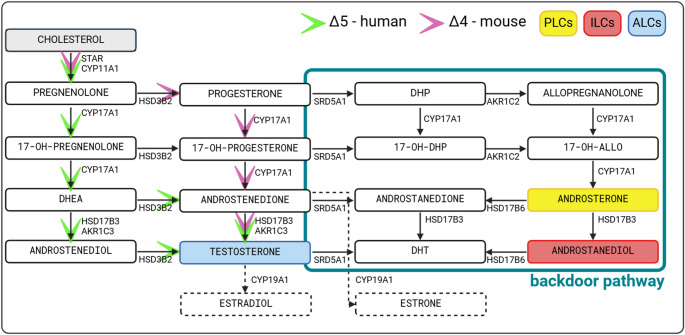
Schematic representation of testicular steroidogenesis, illustrating key human enzymes and androgenic products across distinct Leydig cell developmental stages. Species-specific differences in steroidogenic pathways are highlighted: green arrows indicate the Δ5 pathway predominant in humans, while purple arrows represent the Δ4 pathway typical of rodents.

Existing in vitro models for testicular steroidogenesis have inherent limitations. Most rely on single-cell type cultures, which fail to replicate the complexity and dynamic interactions of human physiology (Parks Saldutti et al. 2013). The human H295R cell line, derived from an adrenal corticocarcinoma of a female patient, remains the only formally validated assay for assessing human steroidogenesis (OECD TG456; OECD 2025). However, its relevance to testicular steroidogenesis is debated due to fundamental differences between adrenal and gonadal cells, female and male physiology, and species-specific pathways (Fig. 2) (del Valle et al. 2017; Habert et al. 2014; Mamsen et al. 2017). Moreover, a limited understanding of steroid hormone production in normal versus cancerous adrenal cells further restricts its applicability for chemical safety assessment (Botteri Principato et al. 2018; Pinto et al. 2018).

In males, Leydig cells, located between the seminiferous tubules in the testes, are the primary source of androgens. Their morphology, marker expression, and steroid hormone profiles vary markedly across developmental stages and aging (Table 1; Fig. 2). Fetal Leydig cells mainly produce androstenedione, which is converted to testosterone by HSD17B3 expressed in fetal Sertoli cells (Li et al. 2022; Martin 2016). Postnatally, fetal Leydig cells are gradually replaced by adult Leydig cells. Within the adult lineage, progenitor Leydig cells (PLCs) predominantly secrete androsterone, immature Leydig cells (ILCs) produce androstanediol, and mature Leydig cells synthesize testosterone (Li et al. 2022).

Steroidogenic regulation is both stage-specific and species-specific (Table 1; Fig. 2). In humans and primates, pregnenolone is preferentially converted to dehydroepiandrosterone (DHEA) via the Δ5 (D5) pathway, with CYP17A1 catalyzing the 17,20-lyase reaction. This preference reflects the enzyme’s higher affinity for 17OH-pregnenolone compared to 17OH-progesterone. In contrast, rodents primarily utilize the Δ4 (D4) pathway, converting pregnenolone to testosterone through progesterone and androstenedione. In this route, CYP17A1 exhibits mainly 17α-hydroxylase activity due to its stronger affinity for 17OH-progesterone (Lawrence et al. 2022; Miller and Auchus 2019).

To effectively study gonadal steroidogenesis, its molecular regulation, and chemical disruption in humans, in vitro models must be of human origin and capture the full spectrum of Leydig cell functions across developmental stages. This review provides a comprehensive evaluation of available in vitro models of testicular steroidogenic cells, highlighting their applications, limitations, and future directions for advancing chemical safety assessment.

**Table 1:**
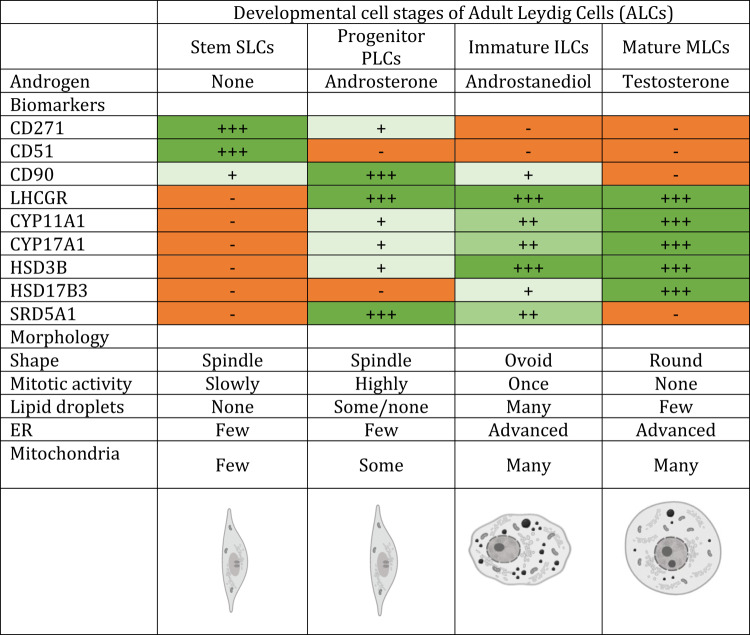
Stage-specific markers and characteristics of human adult Leydig cell types

The table summarizes key androgenic outputs, molecular markers, and morphological features across stem, progenitor, immature, and mature adult Leydig cell stages (Eliveld et al. 2019; Jiang et al. 2014; Li et al. 2022; Svechnikov et al. 2010; Ye et al. 2017). ER: endoplasmic reticulum; LCs: Leydig cells

## Methods–search strategies

### Data collection for in vitro models and related information–search strategy 1

An initial literature search was conducted in PubMed on October 19 and 21, 2022, and updated on November 11, 2024, using the Abstract Sifter tool v6.2 (Baker et al. 2017) to identify studies employing in vitro models for testicular steroidogenesis. Two complementary search strategies were applied: one targeting Leydig-relevant cells and the other focusing on stem cell-based Leydig-like cell models. Detailed search queries are provided in Supplementary Texts S1 and S2.

The 2022 search retrieved 4,592 papers on Leydig-relevant cells and 3,168 on stem cell-based Leydig-like models; the 2024 update added 466 and 334 papers, respectively. Additionally, recent publications on testicular organoids were reviewed (Alves-Lopes and Stukenborg 2018; Bashiri et al. 2023; Cham et al. 2021; Horvath-Pereira et al. 2023; Huleihel and Lunenfeld 2020; Ingber 2022; Kilcoyne and Mitchell 2019; Komeya et al. 2018; Nengzhuang et al. 2022; Richer et al. 2020; Sakib et al. 2019, 2020; Salem et al. 2023). 30 additional primary research papers on in vitro 3D models relevant to Leydig cell steroidogenesis were included. All records were compiled, and duplicates removed. Two reviewers independently screened studies using the Abstract Sifter tool (Baker et al. 2017).

Inclusion criteria were: (1) Mammalian in vitro model; (2) Use of Leydig or adrenal cell types, stem Leydig cells, or Leydig-like cells derived from other cell types (e.g., stem cells, mesenchymal cells, fibroblasts) applied to study steroid (sex) hormone production; and (3) Original research article. Exclusion criteria included: (1) Studies investigating adrenal steroidogenesis without assessing sex hormone production (e.g., focusing exclusively on glucocorticoids or mineralocorticoids); (2) Studies unrelated to steroidogenesis (evaluating, e.g., apoptosis, viability, oxidative stress); and (3) Review articles.

A total of 1533 papers met the inclusion criteria and are summarized in Appendix A, covering publications from 1972 to 2024 (Supplementary Figure S1). In most cases, one study corresponds to one paper; however, if a paper included in vitro models from different species or employed multiple distinct models for testicular steroidogenesis, each was counted as a separate study.

Data were extracted for the following parameters: (1) Agents used to stimulate steroidogenesis; (2) Parameters related to cholesterol transport; (3) Regulatory markers of steroidogenesis; (4) Enzymes involved in testicular steroidogenesis; and (5) Measured hormones. To ensure specificity, only studies utilizing a single Leydig cell type or its alternatives were included (1366 studies). Studies describing more than one relevant model in the abstract were excluded, as semi-automated screening does not allow accurate attribution of parameters to individual models. Keywords for each term were identified using Abstract Sifter and TermMap (Baker et al. 2017). Each study was scored as 1 if the term appeared in the abstract, and 0 if not. For general or ambiguous terms, punctuation or spacing was added to avoid misclassification (Supplementary Table S1). Search strategies were refined to minimize over- or underestimation and validated using a random sample of 75–100 studies per term. The final dataset included 356 studies on primary cells, 41 on stem-cell-derived Leydig-like cells, and 969 on Leydig or adrenal cell lines (H295R: 308; MA-10: 370; TM3: 151; mLTC1: 95; R2C: 45). Term-specific study counts are provided in Supplementary Table S2.

### Data collection for potential reference chemicals–search strategy 2

To evaluate chemical effects on steroid hormone production—testosterone, progesterone, androstenedione, and estrogen (primarily 17β-estradiol)—in in vitro models of male steroidogenesis, a multi-step strategy was applied (Supplementary Figure S2). The initial list of 23 chemicals was derived from those used in the OECD H295R validation study (Hecker et al. 2011), and supplemented with data-rich compounds classified as Group 1 by Pinto et al. (Pinto et al. 2018).

In the first step, papers identified by Pinto et al. (2018) were retrieved from their Supplementary Excel lists. In the second step, the previously compiled Abstract Sifter database (Appendix A) was screened using chemical names, abbreviations, and alternative identifiers. In the third step, targeted PubMed searches were performed for each chemical using Abstract Sifter with the following query: [Chemical name] AND [“Leydig” AND (“cell line” OR “in vitro” OR “ex vivo” OR “primary cell*”) AND (hormone OR androgen OR progesterone OR estrogen)]. Where applicable, alternative names, abbreviations, and CAS numbers were included to broaden the search. Duplicate records across steps were removed, and all entries were screened for inclusion.

In total, 622 papers (818 chemical-paper pairs) were screened, of which 207 were included (Appendix B) based on these criteria: (1) Individual chemical exposure compared to a defined control; (2) Use of a relevant mammalian in vitro model (H295R, Leydig cell models, or advanced testicular models such as testis explants); (3) Measurement of selected hormones (testosterone, progesterone, androstenedione, and estrogen (17β-estradiol)); (4) Original research article (reviews excluded).

Studies conducted under both basal and induced conditions (e.g., stimulation with LH or human chorionic gonadotropin (hCG)) were included, and induction status was recorded (Appendix B). For each chemical, the number of studies per hormone and model was documented based on full-text analysis, along with the proportion of studies reporting an increase, decrease, or no effect. Additional parameters extracted from keyword-based searches included culture conditions, agents used to stimulate steroidogenesis, markers of cholesterol transport, steroidogenic enzymes, and measured hormones. Methods for hormone quantification were also recorded to support cross-study comparisons.

In general, one study corresponds to one paper, with two exceptions resulting in a higher study count than paper count: (1) Multi-laboratory studies (e.g., (Hecker et al. 2011) were counted separately for each participating laboratory; (2) Studies using different cell lines or primary Leydig cells or testis explants from different species or developmental stages were treated as distinct studies. The final dataset included 95 studies on primary Leydig cells, including testis explants or organ cultures, 6 on stem-cell-derived Leydig-like cells, and 141 on Leydig or adrenal cell lines (H295R: 77; MA-10: 32; TM3: 17; mLTC1: 8; BLTK1: 3). Term-specific study counts are provided in Supplementary Table S3.

If a single study reported opposing effects (e.g., concentration-dependent or basal vs. induced conditions), both effects were noted, but the study was counted once. For example, if 2 studies are available—1 reporting an increase and the other reporting both an increase and a decrease—the total count is 2, with 75% reporting an increase and 25% a decrease.

## Results and discussion

### Cell types used for exploring testicular steroidogenesis in vitro

Bouftas et al. (2026) recently introduced an upstream AOP network for disrupted steroidogenesis, emphasizing reduced enzyme activity and hormone production. This framework highlights key enzymes such as CYP11A1, CYP17A1, and HSD3B, which are central to testicular steroidogenesis, and offers opportunities to link molecular events to adverse outcomes. Integrating Leydig cell-specific in vitro models into such networks could improve mechanistic interpretation and regulatory relevance. Our review complements this approach by evaluating models needed to populate these networks with robust data, particularly for male-specific pathways and developmental stages.

**Fig. 3:**
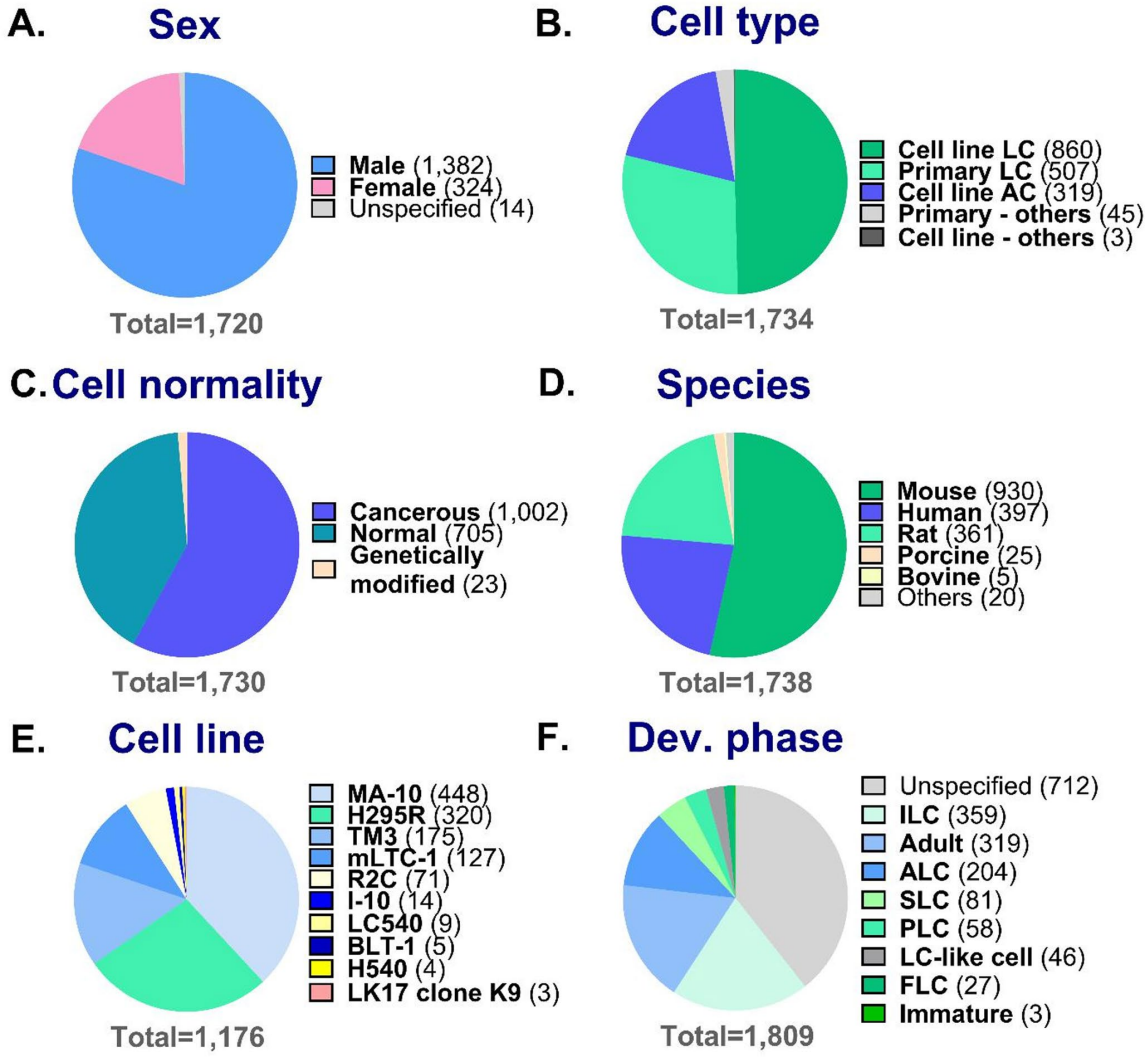
Overview of in vitro models currently used to study testicular steroidogenesis, identified through general keyword-based searches (Search Strategy 1). AC, adrenal cell; F, female; LC, Leydig cells; M, male

Using Search Strategy 1, we analyzed in vitro models employed to study testicular steroidogenesis. Although this process is male-specific, the adrenal H295R cell line, derived from a female carcinoma, remains widely used, representing 19% of models by sex and 18% by cell type (Fig. 3A and B). Most models were based on Leydig cells, including immortalized lines (50%) and primary cells (29%), with only 2.6% using alternative sources such as mesenchymal- or stem-cell-derived Leydig-like cells.

Regarding species origin and cellular characteristics, the majority of models were based on cancerous or genetically modified cells (61%) (Fig. 3C) and were predominantly rodent-derived (mouse: 54%, rat: 21%). Human cells accounted for only 23%, with minor contributions from porcine (1.4%), bovine (0.3%), and other species (1.2%) (Fig. 3D). Among Leydig cell lines, murine MA-10 cells were most frequently used (38%), followed by TM3 (15%), mLTC-1 (11%), rat R2C (6%), and murine I10 cells (1%) (Fig. 3E). The adrenal H295R cell line accounted for 27% of all cell lines analyzed. Of these, only the murine Leydig TM3 cells are of non-cancerous origin, having been spontaneously immortalized (Mather 1980; Mather et al. 1982).

Chemicals, including EDCs, exert particularly strong effects during human development, where they can disrupt androgen signaling and contribute to male reproductive disorders such as cryptorchidism, hypospadias, impaired spermatogenesis, and testicular cancer—primarily through antiandrogenic mechanisms (Klinefelter et al. 2025; Rodprasert et al. 2021; Sharpe 2024; Svechnikov et al. 2010; Yang et al. 2025). Leydig cell characteristics, including morphology, marker expression, and hormone production, vary markedly across developmental stages (Table 1; Fig. 2), underscoring the need to clearly specify the developmental stage of Leydig cells used in in vitro models.

Leydig cells follow two major lineages: fetal and adult. Both originate from stem Leydig cells (SLCs) and progress to PLCs (Chen et al. 2017; Shima 2019). In adult development, progenitors further differentiate into ILCs and mature adult Leydig cells (ALCs). Each stage is characterized by distinct morphological features and progressive maturation of steroidogenic enzymes (Table 1), resulting in stage-specific androgen production (Fig. 2). Fetal Leydig cells in humans and rodents arise from specialized progenitor populations, but do not clearly transition through distinct immature and mature stages as observed in adult Leydig cell development (Dong et al. 2007; Ye et al. 2017).

Despite the complexity of Leydig cell development, most rodent Leydig cell lines, such as MA-10 and mLTC-1, originate from males of undefined age. Among them, Leydig TM3 cells are the only established line derived from a clearly defined developmental stage: 11–13-day-old male mice (Mather 1980). These cells represent a postnatal, prepubertal phase and express markers characteristic of immature Leydig cells (Sychrová et al. 2022; Yawer et al. 2020). While primary Leydig cells can be isolated to reflect specific developmental stages, they are predominantly obtained from rodents (Fig. 3) and rapidly lose their steroidogenic capacity under conventional two-dimensional (2D) culture conditions (Risbridger and Hedger 1992). Immature primary Leydig cells are rarely used to model the full course of Leydig cell development in vitro, and even advanced organotypic culture systems fail to fully recapitulate developmental progression (Moutard et al. 2023). Although primary human Leydig cells would offer the most relevant model for studying human testicular steroidogenesis, their availability, particularly during fetal and immature stages, is extremely limited and further constrained by ethical considerations.

In our dataset, approximately 40% of studies did not report the developmental stage or failed to specify it in the abstract (Fig. 3F). Among those that did, only 2% reflected the fetal lineage, while the remaining studies focused on adult lineage cells: stem (5%, SLCs), progenitor (3%, PLCs), immature (18%, ILCs), and mature (12%, ALCs). An additional 18% were isolated during immature or adult age without further specification. Models based on stem cell-derived Leydig-like cells—including those derived from induced pluripotent stem cells (iPSCs)—accounted for 2%.

**Fig. 4:**
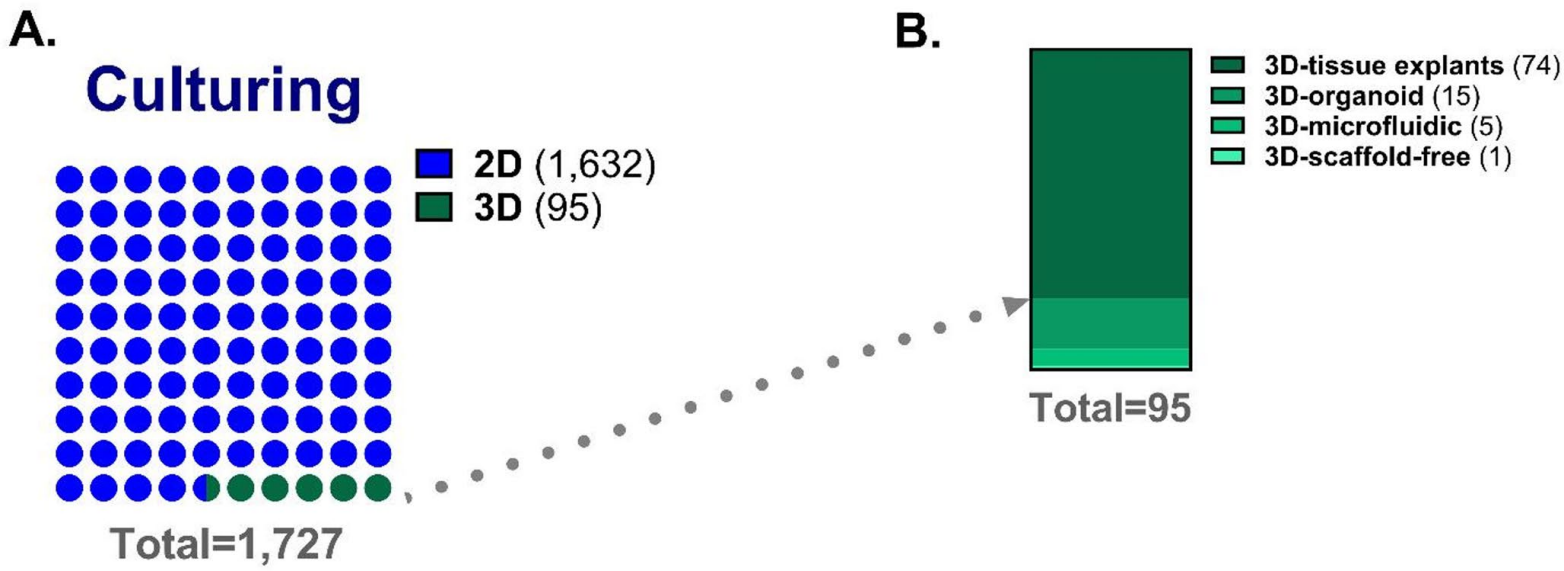
Culture conditions used in in vitro models of testicular steroidogenesis identified through general keyword-based searches (Search Strategy 1).** A** Distribution of conventional two-dimensional (2D) versus three-dimensional (3D) systems (total = 1727 studies).** B** Breakdown of advanced 3D approaches, including tissue explants, organoids, microfluidic systems, and scaffold-free cultures (total = 95 studies). 2D, two-dimensional; 3D, three-dimensional

As in vitro biology and toxicology have advanced, cell culture conditions have become a critical determinant of how accurately these models replicate in situ cellular, tissue, or organ-level physiology. The non-mitotic nature of mature Leydig cells presents challenges for maintenance in conventional 2D cultures, often leading to molecular alterations that impair their steroidogenic function. Our recent findings demonstrated that in a three-dimensional (3D) culture system, Leydig TM3 cells exhibited significantly higher expression of key steroidogenic markers (Sf1 (*NR5A1*), *StAr*, *Cyp11a1*, and *3α-Hsd*), compared to 2D cultures (Sychrová et al. 2022). These results underscore that culture dimensionality alone can enhance the physiological relevance and predictive capacity of in vitro models for testicular steroidogenesis. Nevertheless, the vast majority of in vitro models remain 2D-based (95%). Among 3D models, testicular tissue explants are most commonly employed (78%), while organoid-based systems account for only a small fraction (16%) (Fig. 4**)**.

### Markers and parameters for characterizing testicular steroidogenesis in vitro

**Fig. 5:**
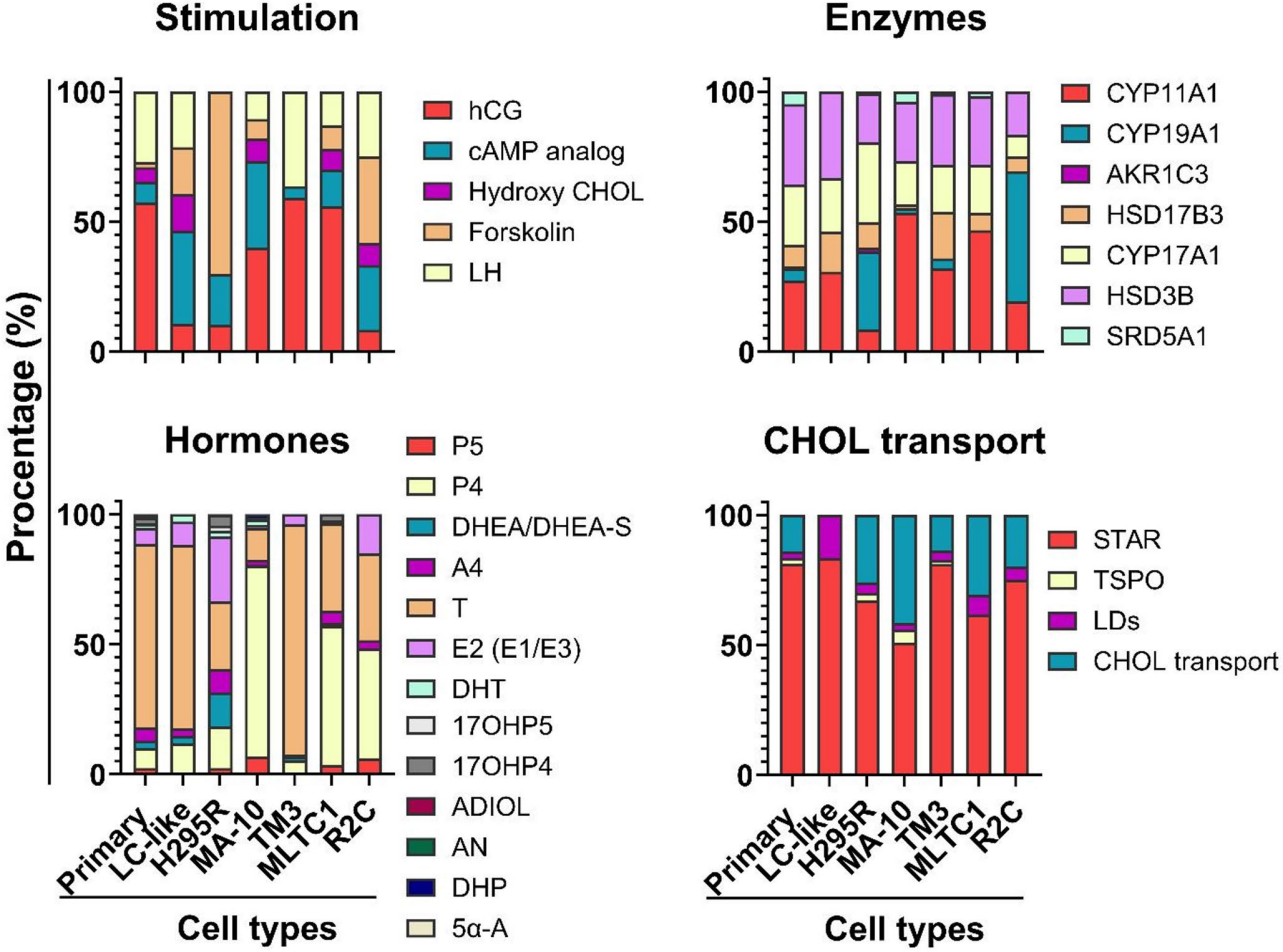
Summary of in vitro studies identified through general keyword-based searches (Search Strategy 1), highlighting agents used to stimulate steroidogenesis, steroidogenic enzymes and hormones evaluated, and parameters assessed for cholesterol transport. The number of studies addressing each parameter is summarized in Supplementary Table S2

As a next step, we conducted a detailed analysis of the included studies, examining agents used to stimulate steroidogenesis, steroidogenic enzymes and hormones assessed, and parameters related to cholesterol transport (Fig. 5; Supplementary Table S2; Appendix A). Analyses were stratified by model type: primary Leydig cells, stem-cell-derived Leydig-like cells, and established cell lines.

The most commonly used stimulants, sometimes used only as positive controls, were hCG, cyclic AMP (cAMP) analogs, and LH, with usage varying by cell type. Primary Leydig cells were predominantly stimulated by hCG or LH, whereas H295R cells, when stimulated, were typically treated with forskolin. Testosterone was the most frequently measured hormone in studies using primary Leydig cells and stem-cell-derived Leydig-like cells. In H295R cells, testosterone was also commonly assessed alongside estrogens (mainly 17β-estradiol), as recommended by OECD TG456 (OECD 2025), and progesterone. In most Leydig cell lines (MA-10, mLTC1, R2C), progesterone was the primary hormone measured, reflecting their suitability for studying early steroidogenic steps up to progesterone synthesis, but not the complete testosterone biosynthetic pathway (Engeli et al. 2018). Only a limited number of studies profiled steroid hormones across the entire testicular steroidogenic cascade in all model types (e.g., Hasegawa et al. (2013) for H295R cells, Engeli et al. (2018) for MA-10, Tsai et al. (2003) for primary Leydig cells, or Tanaka et al. (2007) for stem-cell-derived Leydig-like cells) (Appendix A), which restricts comprehensive pathway analysis.

Expression levels of key steroidogenic enzymes, including CYP11A1, CYP17A1, and HSD3B (Fig. 2), were most frequently evaluated across in vitro models. Aromatase (CYP19A1) was assessed mainly in H295R and R2C cells, as these models are commonly used to study the biotransformation of testosterone to estrogens. Given that cholesterol transport is a critical initiating step in steroidogenesis, we also reviewed studies assessing related parameters, including StAR and TSPO expression, pregnenolone formation, and direct cholesterol or lipid droplet (LD) staining. Among these, StAR was the most frequently studied marker.

Our findings reflect the previously discussed limitations of currently available cell lines for studying testicular steroidogenesis. The H295R cell line, while widely used, constitutively produces steroid hormones and expresses only low levels—or in some reports, lacks expression entirely—of 17β-HSD3, the key enzyme responsible for testicular testosterone synthesis. Consequently, the low concentrations of testosterone detected in H295R cultures often originate from serum supplements or alternative enzymatic pathways rather than from the physiological testicular steroidogenic cascade (Jäger et al. 2023; Strajhar et al. 2017). Bouftas et al. (2026) propose expanding AOP-Wiki content to capture steroidogenic pathways and emphasize the importance of expanding TG456 to include additional steroid hormones. However, our findings highlight that even with such expansion, adrenal-based models cannot fully capture testicular steroidogenesis and its regulation.

Murine Leydig cell lines such as TM3, MA-10, and mLTC-1 also fail to accurately model human testicular steroidogenesis due to species-specific differences in steroidogenic pathways and enzyme expression (Fig. 2). Notably, these cell lines exhibit low or undetectable levels of 17β-HSD3, resulting in limited testosterone synthesis and altered steroid profiles compared to the human testis (Engeli et al. 2018; Roelofs et al. 2015). Consequently, they are suitable only for investigating early steps of steroidogenesis, typically producing progesterone as the final product (Engeli et al. 2018), rather than supporting the complete testosterone biosynthetic pathway. Some lines have also lost responsiveness to LH stimulation. For example, the rat Leydig tumor cell line R2C constitutively expresses StAR protein and produces steroids independently of regulatory cues, in contrast to the tightly controlled steroidogenesis observed in normal human Leydig cells (Jo and Stocco 2004). Moreover, R2C cells lack DAX-1, a known repressor of steroidogenesis, leading to unregulated and non-physiological steroid production.

None of the currently available cell lines fully recapitulate human testicular steroidogenesis. H295R cells, derived from the adrenal cortex, lack key testis-specific enzymes and differ fundamentally in their regulatory mechanisms, reflecting distinctions between adrenal and gonadal steroidogenic pathways (Strajhar et al. 2017). Furthermore, the limited understanding of steroid hormone production in normal versus cancerous adrenal cells constrains the utility of the H295R model for chemical safety assessment (Botteri Principato et al. 2018; Pinto et al. 2018). Similarly, rodent Leydig cell lines exhibit species-specific differences in steroidogenic pathways and enzyme expression, including low or absent 17β-HSD3 activity, resulting in incomplete testosterone synthesis. Therefore, findings derived from these models should be interpreted with caution when extrapolating to human testicular function.

### Chemical assessment using in vitro models of testicular steroidogenesis

To compare the performance of different in vitro models in evaluating chemical effects on testicular steroidogenesis, assess data availability, and identify potential reference compounds for Leydig cell-based assays, we screened the literature and validation reports for 23 candidate chemicals obtained through Search Strategy 2, including those used in the OECD H295R validation study.

From 207 included papers, we identified and characterized 242 studies using individual in vitro models of testicular steroidogenesis based on full-text analysis (Supplementary Figure S3, Appendix B). Primary Leydig cells were the most frequently used model (39%), followed by Leydig cell lines (27%). The H295R cell line or its derivatives accounted for 54% of all cell line-based studies, primarily because our chemical set included compounds assessed during the OECD validation of this assay. Murine Leydig cell lines (MA-10, TM3, mLTC-1, BLTK1) represented 43% of cell line-based studies.

Models were nearly evenly split between normal cells (mostly primary) and cancerous cells (predominantly cell lines). The most commonly used species were human, mouse, and rat, with H295R being the predominant human-based model. A wide range of developmental stages was represented, although adult and unspecified stages were most frequent. Developmental identity was often under-characterized; however, the proportion of studies with unspecified stages was lower in the full-text-based analysis compared to the abstract-driven search. This difference may reflect a bias introduced by the search strategy, but it could also indicate that developmental stage information is not systematically reported in abstracts, suggesting that the abstract-based scan slightly overestimates the proportion of unspecified stages. Overall, early developmental stages remained underrepresented.

We also summarized key experimental details, including agents used to stimulate steroidogenesis, steroidogenic enzymes and hormones evaluated, and parameters related to cholesterol transport (Supplementary Figure S4, Table S3, Appendix B). The most commonly used stimulants—sometimes applied only as positive controls—were forskolin (especially in H295R cells), hCG, and LH (primarily in primary cell models). Testosterone was the most frequently measured hormone across most cell types, except in MA-10 cells, where progesterone predominated. Few studies assessed both testosterone and progesterone together with expression of key steroidogenic enzymes (CYP11A1, CYP17A1, HSD3B) or cholesterol transport markers, with StAR being the most frequently evaluated. Another common limitation was the narrow scope of hormone measurements, typically covering only one to three hormones, which restricts comprehensive profiling of the steroidogenic pathway. Most in vitro models were cultured under 2D conditions (89%). Among 3D systems, rodent-derived testicular explants or organ cultures were most common. Hormone detection and quantification relied primarily on immunoanalytical techniques (85%), while mass spectrometry was used in only a limited number of studies (Supplementary Figure S5, Appendix B).

**Fig. 6:**
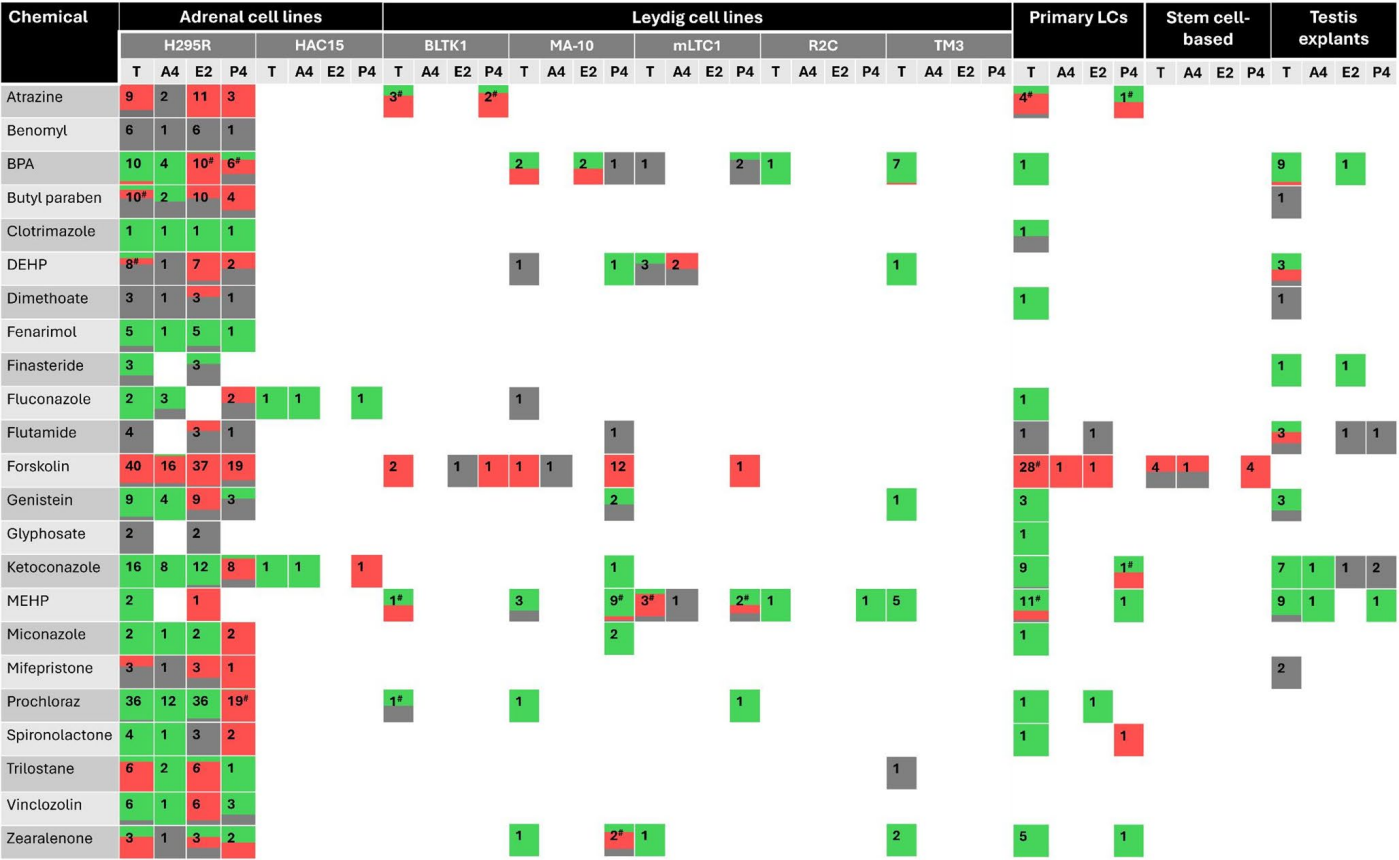
Comparison of the effects of 23 selected chemicals on hormone levels during testicular steroidogenesis, evaluated in in vitro models identified through Search Strategy 2. The figure shows the number and proportion of studies reporting a decrease (green), increase (red), or no effect (dark grey) on testosterone (T), androstenedione (A4), estradiol (E2), or progesterone (P4) across 9 in vitro models. Opposing effects within a single study (#) reflect differences in concentration or experimental conditions (induced vs. basal). For trilostane-induced effects on T and E2 in H295R cells, italicized numbers indicate that 5 reports originate from multi-laboratory validation studies, where antibody cross-reactivity was noted as a concern

Having characterized the models and their experimental setups, we now turn to the selected 23 chemicals and their effects on these systems. From the 401 individual chemical–study pairs (Appendix B), Fig. 6 summarizes the number of studies evaluating specific hormones—testosterone, progesterone, androstenedione, and estrogens (17β-estradiol unless otherwise specified)—across different models and chemicals, as well as the observed effect of each chemical on hormone levels.

**Fig. 7:**
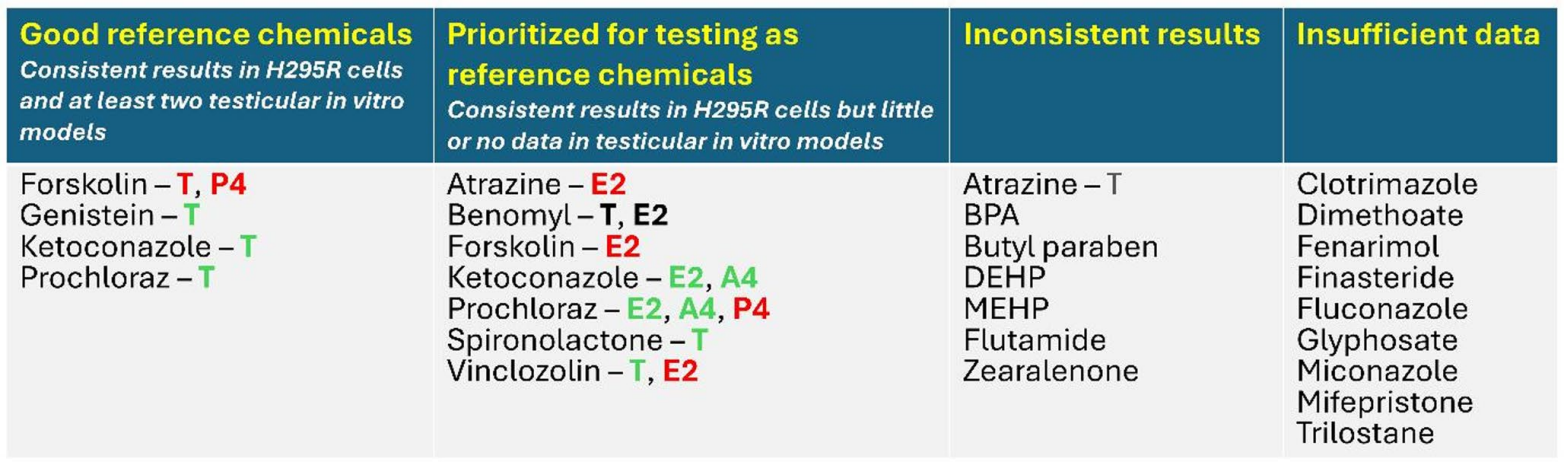
Proposed reference chemicals for in vitro testicular steroidogenesis models, categorized by hormone-specific effects (increase–red, decrease–green) based on the number and consistency of studies reporting changes in steroid hormone levels. “Good reference” chemicals consistently affected the indicated hormone(s) in at least five studies, including data from a minimum of two gonadal models. Chemicals “prioritized for testing” showed consistent effects in at least five studies but lacked sufficient supporting data from gonadal models

Chemicals were categorized based on the consistency of hormone-specific effects in steroidogenesis assays and the availability of data across H295R and testicular in vitro models (Figs. 6 and 7). The 23 compounds span diverse classes and chemical structures, including pharmaceuticals (e.g., ketoconazole, spironolactone, trilostane), pesticides (e.g., atrazine, vinclozolin, prochloraz), plasticizers (e.g., DEHP-di-(2-ethylhexyl) phthalate and its metabolite MEHP-mono-(2-ethylhexyl) phthalate), and phytoestrogens (e.g., genistein), and reflect varied mechanisms of action on steroidogenic enzymes and hormone receptors.

Four compounds— forskolin (a diterpene from *Coleus forskohlii*), genistein (an isoflavone from soy), prochloraz (an imidazole fungicide), and ketoconazole (an antifungal drug)—showed consistent effects on testosterone across H295R and at least two testicular models, making them strong candidates as reference compounds. Forskolin also increased progesterone across models. In addition, forskolin, ketoconazole, and prochloraz affected 17β-estradiol; ketoconazole and prochloraz influenced androstenedione, and prochloraz also impacted progesterone, mainly in H295R cells.

Atrazine (a widely used triazine herbicide), benomyl (a systemic benzimidazole fungicide), spironolactone (a synthetic steroidal diuretic), and vinclozolin (a dicarboximide fungicide) produced reproducible effects on testosterone and/or estradiol in H295R but lacked sufficient Leydig-based data, warranting prioritized testing. In contrast, bisphenol A (BPA, an industrial chemical used in plastics), DEHP and its metabolite MEHP (phthalate plasticizers), butyl paraben (a synthetic preservative), flutamide (a non-steroidal anti-androgen), and zearalenone (mycotoxin) showed inconsistent results for testosterone or other hormones, limiting their reliability as reference compounds.

Finally, 9 chemicals remain largely untested in both H295R and testicular models, representing critical data gaps. These include azole antifungals (clotrimazole, fluconazole, miconazole), organophosphate insecticides (dimethoate), pyrimidine fungicides (fenarimol), steroidogenesis inhibitors (finasteride, trilostane), herbicides (glyphosate), and synthetic steroidal modulators (mifepristone).

Detailed evaluation of individual chemicals (Fig. 6, Appendix B) revealed that forskolin consistently increased testosterone and progesterone across diverse in vitro model, including H295R cells (e.g., Hecker et al. (2006; Liu et al. (2012); Mangelis et al. (2016), Leydig cell lines (MA-10, mLTC1, BLTK-1; e.g., Engeli et al. (2018); Forgacs et al. (2012); Stocco and Chaudhary (1990), primary Leydig cells from several species and developmental stages (e.g., Ling Poon et al. (2005); Valenti et al. (1995), and stem cell-derived Leydig-like cells (e.g., Yang et al. (2020, 2014). Testosterone elevation was reported in 67 of 75 studies, and progesterone in 34 of 38 studies, with effective concentrations typically 0.1–100 µM (EC_50_: 0.3–3 µM). Forskolin also increased 17β-estradiol in 33 of 35 H295R studies (e.g., Hecker et al. (2011, 2006); LeBaron et al. (2014), though only two male gonadal models showed similar effects (Forgacs et al. 2012; Mankidy et al. 2014), with effective concentrations 0.01–10 µM (EC_50_: 0.6–4 µM). Androstenedione rose in 13 of 16 H295R studies, with effective concentrations ≥ 0.05 µM (e.g., Higley et al. (2010); Rijk et al. (2012); Running et al. (2022); Weisser et al. (2016), but data in gonadal models were limited and inconsistent: no effect in MA-10 cells (Engeli et al. 2018), inconsistent effects in stem cell-based models (Eliveld et al. 2019), and an increase in adult rat primary Leydig cells (Valenti et al. 1997).

Genistein consistently decreased testosterone in 8 of 9 H295R studies and androstenedione in all 4, while increasing estradiol in most cases, with effective concentrations 0.5–50 µM (e.g., Hecker et al. (2011); Nielsen et al. (2012); Tinwell et al. (2013). Comparable inhibitory effects were observed in Leydig cell lines (Yang et al. 2022), rodent primary Leydig cells (Akingbemi et al. 2007; Jeminiwa et al. 2020; Napier et al. 2014), and testis explants, with sensitivity varying by developmental stage (Freyberger et al. 2010; Lehraiki et al. 2011).

Prochloraz robustly inhibited testosterone (34 of 36 studies), androstenedione (12 of 12), and 17β-estradiol (32 out of 36 studies) while consistently inducing progesterone (19 of 19, with one study reporting induction only at lower concentrations) in H295R cells. Effective concentrations ranged from 0.01 to 100 µM (e.g., Haggard et al. (2018); Hecker et al. (2011); Rijk et al. (2012); Weisser et al. (2016). Comparable testosterone inhibition was observed in several gonadal models; however, one BLTK-1 study reported no effect under basal conditions (Forgacs et al. 2012; Mankidy et al. 2014; Roelofs et al. 2014).

Ketoconazole consistently suppressed testosterone (16 of 16 studies), androstenedione (8 of 8), and 17β-estradiol (11 of 12) in H295R cells (e.g., Haggard et al. (2018); Hecker et al. (2011); Rijk et al. (2012); van der Pas et al. (2012); Weisser et al. (2016) and testosterone in Leydig cell-based models, including primary Leydig cells (e.g., Fort et al. (1995); Hanger et al. (1988); Kan et al. (1985) and in fetal human and rat testis explants (e.g.,Freyberger et al. (2010); Powlin et al. (1998). Effective concentrations ranged from 0.1 to 20 µM. Progesterone responses varied among studies, possibly influenced by assay methodology (e.g., antibody cross-reactivity with pregnenolone in immunoassays versus mass spectrometry) and concentration-dependent effects, with increases at low concentrations and decreases at higher concentration (Brun et al. 1991; Rijk et al. 2012).

Benomyl appears to be a reliable negative reference chemical for testosterone and 17β-estradiol based on H295R data (Hecker et al. 2011). Atrazine (a triazine herbicide), spironolactone (a steroidal diuretic), and vinclozolin (a dicarboximide fungicide) also show promise as reference inhibitors of testosterone and/or activators of 17β-estradiol (Hecker et al. 2006, 2011; Jäger et al. 2023; Maglich et al. 2014; van Ravenzwaay et al. 2013), but require validation in testicular models. Atrazine illustrates variability seen for other hormones. In H295R cells, it increased testosterone in 7 of 9 studies and progesterone in all 3 (e.g., Hecker et al. (2011), with similar trends in 3 Leydig-based studies (Forgacs et al. 2013; Kucka et al. 2012; Pogrmic-Majkic et al. 2010). However, two H295R studies found no effect (Hecker et al. 2011), and several Leydig studies reported inconsistent or decreased testosterone under induced conditions (Forgacs et al. 2012; Friedmann 2002; Karmaus and Zacharewski 2015). Androstenedione and estradiol were not assessed in Leydig models, limiting the reliability of atrazine as a reference compound for these two hormones.

BPA, DEHP, MEHP, butyl paraben, flutamide, and zearalenone also show substantial inconsistencies. BPA typically decreased testosterone (9 of 10 studies) and androstenedione (4 of 4) while increasing estradiol (9 of 10) in H295R cells (Kolle et al. 2012; Nielsen et al. 2012). In testicular models, testosterone was reduced in most studies (17 of 21), including human and rodent testis explants (e.g., Akingbemi et al. (2004); Lan et al. (2017); Maamar et al. (2015); N’Tumba-Byn et al. (2012), though 3 studies reported increases (Chen et al. 2018; Dankers et al. 2013; Kolle et al. 2012), one reported no effect (Roy et al. 2023), or inconsistent outcomes (Jambor, 2023). Similar variability was noted in animal studies (Ortega-García et al. 2021). Epidemiological data generally link BPA exposure to increased total testosterone but decreased free testosterone in men (Lü et al. 2024). Other hormones were rarely assessed in Leydig-based models.

DEHP and its metabolite MEHP yielded inconsistent results across studies. Approximately half of the H295R studies reported no effect on testosterone level (e.g., Haggard et al. (2018); Hecker et al. (2011), while others noted increases, decreases, or concentration-dependent changes (Desdoits-Lethimonier et al. 2012; Duan et al. 2020; Moche et al. 2021). Outcomes were similarly variable across 51 Leydig‑based models (e.g., Chauvigné et al. (2009); Desdoits-Lethimonier et al. (2012); Romain et al. (2009); Stroheker et al. (2006); Svechnikov et al. (2008); Zhao et al. (2012), and epidemiological studies show the same pattern of inconsistency (Mínguez-Alarcón et al. 2023; Radke et al. 2018). In H295R cells, 17β-estradiol levels generally increased after exposure to DEHP/MEHP (e.g. Hecker et al. (2011), but this endpoint was not assessed in most Leydig‑based models.

Butyl paraben generally showed no effect on testosterone or 17β-estradiol in most H295R studies and one rat testicular explant study (Freyberger et al. 2010; Hadrup et al. 2013; Haggard et al. 2018; Hecker et al. 2011; Taxvig et al. 2008), though other reports noted increases (Hecker et al. 2011), decreases (Wang et al. 2014), or concentration-dependent effects(Liang et al. 2023). Other hormones were studied rarely. Flutamide had no effect on testosterone in H295R studies (e.g., Haggard et al. (2018); Hecker et al. (2011) but showed inconsistent outcomes in Leydig-based models, including rat testis explants (e.g., Pardyak et al. (2016); Powlin et al. (1998). It also did not affect 17β-estradiol or progesterone in either H295R or Leydig-based models (e.g., Adamsson et al. (2008); Haggard et al. (2018); Maglich et al. (2014); Pardyak et al. (2016). Zearalenone consistently decreased testosterone in all 9 testicular model studies (primary Leydig cells, TM3, mLTC1, MA-10) (e.g., Eze et al. (2019); Lin et al. (2015); Yang et al. (2007); Zhao et al. (2021); Zhou et al. (2018) but increased it in 2 of 3 H295R studies (Haggard et al. 2018; Kolle et al. 2012).

While a detailed analysis of the sources of these inconsistencies is beyond the scope of this review, heterogeneity in experimental setups—particularly regarding stimulation protocols (e.g., LH or hCG)—is likely a major contributor. Several studies have demonstrated that chemical responses (e.g., to MEHP) vary markedly depending on whether stimulation is applied (Forgacs et al. 2012; Jones et al. 2016). For chemicals such as BPA, MEHP, and zearalenone, discrepancies may also arise from model choice. For example, inconsistencies between H295R and Leydig cell models are evident for fadrozole, a selective aromatase inhibitor not included among the 23 screened chemicals. Fadrozole typically increases testosterone and decreases 17β-estradiol synthesis in rodent Leydig cell studies (Bilińska et al. 1997, Kmicikiewicz and Bilińska 1997a, b), yet paradoxically inhibits not only 17β-estradiol but also androstenedione and testosterone synthesis in H295R cells (Hecker et al. 2006, 2011; Kmicikiewicz and Bilińska 1997a, b; Kudoh et al. 1995). These findings suggest that fadrozole may influence enzymes upstream of aromatase and activate distinct compensatory pathways depending on cell type or species.

It is important to note that H295R cells, derived from a human adrenal carcinoma, not gonadal tissue, lack a fully functional LHR/LHCGR signaling pathway. They do not respond to LH or hCG stimulation, which is why forskolin or cAMP analogs are used to activate steroidogenesis in this model. As a result, chemicals, including EDCs acting via LHR/LHCGR-mediated pathways (e.g., LH mimetics or antagonists such as benzo[a]pyrene (Lazzaretti et al. 2024) or DDT (dichlorodiphenyltrichloroethane) (Munier et al. 2021), will show little or no effect in H295R cells, making them unsuitable for assessing mechanisms dependent on gonadotropin signaling.

Furthermore, hormone-controlled culture systems are essential for investigating steroidogenic regulation while minimizing confounding effects from undefined serum factors. Most studies assessed chemical exposures in H295R cells using low concentrations of Nu serum in accordance with OECD TG456 (OECD 2025), and in serum-free or hormone-depleted media for Leydig cell-based models. However, these conditions were often applied without prior cell adaptation, even though cells were typically maintained in serum-containing media (Appendix B). The presence of hormones in serum, even at low levels, or abrupt transfer from serum-containing to serum-free or depleted conditions without adaptation complicates mechanistic interpretation and may contribute to inconsistent results (e.g., for BPA or phthalates such as DEHP, Figs. 6 and 7).

Across all cell models, including H295R cells, chemical effects on androstenedione or progesterone production remain underreported, and few studies address 17β-estradiol levels in male gonadal in vitro models. Consequently, robust reference chemicals for these hormones cannot currently be identified. Ketoconazole, which appears to inhibit androstenedione production in HAC15 cells and testicular explants—likely through suppression of 17,20-lyase (CYP17A1) and aromatase activity (Weber et al. 1989, 1991)—is a potential candidate, but data remain limited. Given these gaps, direct comparison of various steroidogenesis models is not feasible, and their relative reliability remains difficult to assess.

## Study limitations

This review employed a semi-systematic approach, with statistical summaries primarily derived from abstracts and supported by selective full-text screening, particularly for identifying potential reference chemicals. The literature search was limited to the PubMed database, which may have resulted in the omission of studies indexed elsewhere. Nevertheless, given the large number of included studies (> 1500), we consider the observed trends and proportions robust and unlikely to change substantially with additional sources.

No formal quality assessment criteria were applied, as the scope and volume of the literature made such an evaluation impractical. Instead, a basic inclusion criterion—namely, the presence of a control group—was used to ensure a minimum level of experimental rigor.

Both induced and non-induced studies (e.g., LH or hCG stimulation) were included. Although these conditions were not distinguished in Fig. 6, they were reported in the summary table of potential reference chemicals (Appendix B) and discussed in relation to their impact on testicular steroidogenesis. This approach allowed broader coverage but may have introduced variability in reported effects. Future reviews should consider stratifying studies by induction status to better elucidate its influence on chemical responses.

## Future perspectives and challenges

A more comprehensive functional comparison between the H295R adrenal cell model and testicular in vitro models is needed to evaluate their respective performance in chemical safety assessment. This should begin by focusing on chemicals that are already well characterized in H295R but lack sufficient data in testicular in vitro models. Such comparisons should incorporate advanced models and stratify findings by cell type (adrenal vs. gonadal), sex (male vs. female), developmental stage, and species specificity. Despite their promise, most available models are from rodent origin and lack formal validation and OECD regulatory acceptance, raising concerns about their relevance for human risk assessment—particularly given genomic differences and species-specific variations in steroidogenesis pathways (Habert et al. 2014) (Fig. 2).

A major limitation of commonly used Leydig cell lines is their incomplete steroidogenic capacity. Many lack HSD17B3, an enzyme essential for the final step of testosterone synthesis (Engeli et al. 2018). In addition, most lines do not express a fully functional LHR/LHCGR, compromising their responsiveness to physiological stimulation. Leydig cell lines such as TM3, MA-10, and mLTC-1 often exhibit low or declining endogenous LHR expression, resulting in diminished LH responsiveness—a recognized limitation for in vitro studies. Even primary Leydig cells cultured under conventional 2D conditions show a progressive decline in LH responsiveness compared to freshly isolated cells. When LHR is present, mutations or altered receptor trafficking can reduce cell surface expression or impair coupling efficiency, further limiting LH-induced cAMP production and steroidogenesis (El-Hefnawy and Huhtaniemi 1998; Engeli et al. 2018; Hirakawa et al. 2002; Koganti et al. 2022).

Bouftas et al. (2026) recommend incorporating hormone ratios and alternative pathways—such as the backdoor route to dihydrotestosterone (DHT) synthesis—into AOP networks. These concepts align with our recommendation to develop advanced Leydig cell models that capture the full complexity of steroidogenesis and support mechanistic endpoints beyond single hormone measurements. To improve the predictive value of in vitro testicular steroidogenesis models, future efforts should focus on:

- Developing and validating models with complete steroidogenic pathways.
- Incorporating hormone-controlled culture systems to reduce variability from serum components.
- Establishing standardized protocols for induction and endpoint measurement.
- Enhancing cross-species and developmental stage comparisons to better reflect human physiology.

Ultimately, integrating well-characterized, mechanistically relevant models into regulatory frameworks will be essential for advancing non-animal approaches in EDC testing.

## Supplementary Information

Below is the link to the electronic supplementary material.

Supplementary Material 1

Supplementary Material 2

Supplementary Material 3
