# Supplementary material for: In vitro models for testicular steroidogenesis: current status and future perspectives
Source: Arch Toxicol. 2026 Apr 28;100(8):3211–29. doi: 10.1007/s00204-026-04380-5 (PMC13379425; doi:10.1007/s00204-026-04380-5)
Supplement: Supplementary file 1 — Supplementary Material 1 [file 204_2026_4380_MOESM1_ESM.pdf]

SUPPLEMENTARY MATERIALS

In vitro models for testicular steroidogenesis: Current status and future perspectives

Eliška Řehůřková, Lola Bajard, Iva Sovadinová*

RECETOX, Faculty of Science, Masaryk University, Kotlarska 2, 611 37 Brno, Czech Republic

*iva.sovadinova@recetox.muni.cz, ORCID: https://orcid.org/0000-0003-0627-243X

SUPPLEMENTARY TEXTS

Supplementary Text S1: Search strategy for identifying Leydig cells and other cell types used in *in vitro* studies of testicular steroidogenesis

PubMed query run (19.10.2022 & 11.11.2024): ("H295R" [tw] OR "NCI-H295R" [tw] OR "I-10" [tw] OR "B-1-A-2" [tw] OR "BLT-1" [tw] OR "I-10/DDPh" [tw] OR "I-10/DDPi" [tw] OR "LC-540" [tw] OR "LK17" [tw] OR "MA-10" [tw] OR "K9" [tw] OR "MLTC-1" [tw] OR "R2C" [tw] OR "TM3" [tw] OR "I 10" [tw] OR "LC540" [tw] OR "LC 540" [tw] OR "MA10" [tw] OR "mLTC-1" [tw]OR "R 2 C" [tw] OR "TM-3" [tw] OR "Primary Leydig" [tw] OR "Primary testicular cell" [tw] OR "Primary mice Leydig" [tw] OR "Primary human Leydig" [tw] OR "Isolated Leydig" [tw] OR "Isolated testicular cell" [tw] OR "Isolated mice Leydig" [tw] OR "Isolated human Leydig" [tw]) and ("cell" [tw] OR "cells" [tw] OR "line" [tw])

Supplementary Text S2: Search strategy for identifying stem cell-based *in vitro* models used to study testicular steroidogenesis

Searching strategy for stem cell-based *in vitro* models for testicular steroidogenesis involved two PubMed query runs (A & B). The results from both queries were initially compiled, and afterward, duplicates were removed.**PubMed query run A: (21.10.2022 & 11.11.2024):** (fibroblast [tw] OR "fibroblasts" [tw]) AND (("Testis"[Mesh] OR "Testis" [tw] OR "Testicle" [tw] OR "Testes" [tw] OR "Steroidogenic cells"[tw] OR "male gonadal cells"[tw] OR "Leydig"[tw] OR "Leydig Cells/metabolism"[Mesh] OR "Testicular cells"[tw]OR "Gonadal Lineage"[tw]or "testicular"[tw] OR "male gonad"[tw]) AND ("Gonadal Steroid Hormones"[Mesh] OR "Gonadal Steroid Hormones"[tw] OR "Sex steroid hormones" [tw] OR "Sex hormone" [tw] OR "Sex hormones" [tw] OR "Steroid hormones"[tw] OR "Progesterone Congeners"[Mesh] OR "Pregnenolone"[Mesh] OR "Pregnenolone" [tw] OR "17-alpha-Hydroxypregnenolone"[Mesh] OR "17-Hydroxypregnenolone" [tw] OR "Progesterone"[Mesh] OR "Progesterone"[tw] OR "Testosterone Congeners"[Mesh] OR "Androgens" [tw] OR "Androgen" [tw] OR "Androstane-3,17-diol"[tw] OR "Androstenediol"[tw] OR "Androstenedione"[tw] OR "Androsterone"[tw] OR "Dihydrotestosterone"[tw] OR "Testosterone"[tw] OR "Dehydroepiandrosterone"[tw] OR "steroidogenesis" [tw] OR "Steroidogenic"[tw] OR "Spermatogenesis"[Mesh] OR "Spermatogenesis"[tw] OR "Hypogonadism"[Mesh] OR"Hypogonadism"[tw] OR "Infertility, Male"[Mesh] OR "Infertility, Male"[tw] OR"sex development"[tw]OR"male fertility"[tw]))

PubMed query run B: (21.10.2022 & 11.11.2024): ("Induced Pluripotent Stem Cells"[Mesh] OR " IPSC" [tw] OR " IPSCs" [tw] OR "induced pluripotent stem cell" [tw] OR "induced pluripotent stem cells" [tw] OR "hiPSC" [tw] OR "hiPSCs" [tw] OR "Adult stem cells" [Mesh] OR "Stem cells/metabolism" [Mesh] OR "Adult stem cells" [tw] OR "Stem cell" [tw] OR "Stem cells" [tw] OR "Fetal Stem Cells"[Mesh] OR "Fetal Stem Cells"[tw] OR "Embryonic Stem Cells"[Mesh] OR "Embryonic Stem Cells"[tw] OR "Human Embryonic Stem Cells"[Mesh] OR "Human Embryonic Stem Cells"[tw] OR "Mouse Embryonic Stem Cells"[Mesh] OR "Mouse Embryonic Stem Cells"[tw] OR "Mesenchymal Stem Cells"[Mesh] OR "Mesenchymal Stem Cells"[tw] OR "Marrow Stromal Cells" [tw] OR "Stem Leydig cells " [tw]OR "bone marrow mesenchymal cells"[tw]) AND (("Testis"[Mesh] OR "Testis" [tw] OR "Testicle" [tw] OR "Testes" [tw] OR "Steroidogenic cells"[tw] OR "male gonadal cells"[tw] OR "Leydig"[tw] OR "Leydig Cells/metabolism"[Mesh] OR "Testicular cells"[tw]OR "Gonadal Lineage"[tw]or "testicular"[tw] OR "male gonad"[tw]) AND ("Gonadal Steroid Hormones"[Mesh] OR "Gonadal Steroid Hormones"[tw] OR "Sex steroid hormones" [tw] OR "Sex hormone" [tw] OR "Sex hormones" [tw] OR "Steroid hormones"[tw] OR "Progesterone Congeners"[Mesh] OR "Pregnenolone"[Mesh] OR "Pregnenolone" [tw] OR "17-alpha-Hydroxypregnenolone"[Mesh] OR "17-Hydroxypregnenolone" [tw] OR "Progesterone"[Mesh] OR "Progesterone"[tw] OR "Testosterone Congeners"[Mesh] OR "Androgens" [tw] OR "Androgen" [tw] OR "Androstane-3,17-diol"[tw] OR "Androstenediol"[tw] OR "Androstenedione"[tw] OR "Androsterone"[tw] OR "Dihydrotestosterone"[tw] OR "Testosterone"[tw] OR "Dehydroepiandrosterone"[tw] OR "steroidogenesis" [tw] OR "Steroidogenic"[tw] OR "Spermatogenesis"[Mesh] OR "Spermatogenesis"[tw] OR

"Hypogonadism"[Mesh] OR"Hypogonadism"[tw] OR "Infertility, Male"[Mesh] OR "Infertility, Male"[tw] OR"sex development"[tw]OR"male fertility"[tw]))

SUPPLEMENTARY TABLES

Supplementary Table S1: Overview of keywords and key terms used to identify relevant studies from abstracts, including additional characterization in the context of testicular steroidogenesis.

Search Term		Keywords/ Key terms	Advanced strategy to overcome limitations
STIMULATION	**hCG**	human chorionic gonadotropin<br>chorionic gonadotropin<br>chorionicgonadotropin<br>hCG<br>CG<br>gonadotrophin<br>gonadotrophic hormone<br>gonadotropin<br>gonadotropic hormone	•hCG: This term also captures the name of the receptor hCGLHR. Receptors were filtered by comparing the terms for the receptor. If the term was found both as hCG and LHR, the abstract was manually searched.<br>•hCG usage: hCG may not always be used as a stimulant; it might be included for a basic introduction to a study. Therefore, the abstracts of some studies (too many to be searched manually) were checked manually. Rarely was hCG binding confirmed. Most studies used hCG as a stimulant of steroidogenesis.
	**LH**	lutein<br>LH<br>LH,<br>LH.<br>LH:<br>(LH)<br>LH)<br>LH/<br>lutropin<br>lutrophin	•LH: This term also captures the name of the receptor hCGLHR. Receptors were filtered by comparing the terms for the receptor. If the term was found both as LH and LHR, the abstract was manually searched.<br>•LH usage: LH may not always be used as a stimulant; it might be included for a basic introduction of a study. Therefore, the abstracts of all studies were checked manually and such studies were excluded.
	**cAMP analog**	8-bromo-cAMP<br>(Bu)2cAMP<br>Br-cAMP<br>8-bromo cyclic adenosine monophosphate<br>Bt2cAMP<br>dibutyl cAMP<br>8-br-cAMP<br>8Br-cAMP<br>8-bromo(Br)-cAMP<br>8-bromoadenosine 3′,5′-cyclic monophosphate<br>dibutyryl-cAMP<br>dbcAMP<br>8-Bromo Cyclic Adenosine Monophosphate<br>8-bromoadenosine 3',5'-cyclic monophosphate<br>Bt(2)cAMP<br>[(Bu)(2)cAMP]<br>(Bu)(2)cAMP<br>db-cAMP<br>dibutyryl cAMP<br>dibutyryl [(Bu)(2)]cAMP<br>8bromo-cAMP	•Searched only for synthetic cAMP analogs used for stimulation.<br>•We could not do a brief search using just "cAMP" as it would have included introductions and measurements of cAMP levels. Consequently, we may have missed a few papers that only report cells being stimulated with cAMP. However, cAMP is quite unstable, which is why most studies use cAMP analogs to stimulate steroidogenesis.
	**Hydroxy cholesterol**	22(R)OH-cholesterol<br>22R-hydroxycholesterol<br>hydroxycholesterol<br>25-hydroxycholesterol<br>22-hydroxycholesterol<br>22(R)-hydroxycholesterol<br>22(R)OH cholesterol<br>25-hydroxy cholesterol	•The basic search strategy was sufficient.

		25-OHC	
	**Forskolin**	forskolin<br>colforsin<br>Coleonol	•The basic search strategy was sufficient.
**ENZYMES**	**CYP11A1**	CYP11A1<br>CYP11<br>CYP11.<br>CYP11,<br>(CYP11)<br>CYP11:<br>P450SCC<br>Cytochrome P450 11A1<br>Cytochrome P450(Scc)<br>Cytochrome P450 Family 11<br>P450(scc)<br>cholesterol side-chain cleavage enzyme<br>P450 side-chain cleavage enzyme<br>side-chain cleavage enzyme	•The basic search strategy was sufficient.
	**CYP17A1**	CYP17A1<br>CYP17<br>CYP17,<br>CYP17.<br>(CYP17)<br>CYP17:<br>P450C17<br>cytochrome P450 Family 17<br>Steroid 17-Alpha-Hydroxylase<br>P450-17α<br>cytochrome P450 17α-hydroxysteroid dehydrogenase<br>P450c17	•The basic search strategy was sufficient.
	**CYP19A1**	CYP19A1<br>CYP19<br>CYP19,<br>CYP19.<br>(CYP19)<br>CYP19:<br>Aromatase<br>P-450AROM<br>ARO<br>ARO,<br>ARO.<br>ARO)<br>ARO(<br>(ARO)<br>ARO:<br>Cytochrome P450 Family 19<br>Cytochrome P450 19A1<br>P450arom	•The basic search strategy was sufficient.
	**AKR1C3**	HSD17B5<br>AKR1C3<br>Aldo-Keto Reductase Family 1 Member C3<br>3-Alpha-Hydroxysteroid Dehydrogenase<br>17-Beta-HSD 5	•The basic search strategy was sufficient.
	**HSD17B3**	HSD17<br>HSD17.<br>HSD17,<br>(HSD17)<br>HSD17:<br>HSD17B3<br>HSD17B1<br>SDR12C2<br>17-Beta-HSD<br>17β-HSD<br>Hydroxysteroid 17-Beta Dehydrogenase 3<br>Hydroxysteroid Dehydrogenase 3	•The basic search strategy was sufficient.

ENZYMES		17βHSD<br>17beta-HSD<br>17-βHSD<br>17betaHSD1<br>17beta-HSD1<br>HSD17β1	
	**HSD3B**	HSD3B2<br>3β-HSD<br>SDR11E2<br>HSD3B1<br>HSDB3B<br>Beta-HSD<br>SDR11E1<br>HSDB3A<br>3 Beta-Hydroxysteroid Dehydrogenase<br>3 Beta-HSD Type II<br>HSD3B<br>HSD3B.<br>HSD3B,<br>(HSD3B)<br>HSD3B:<br>Hsd3β<br>3βHSD2<br>3β-HSD<br>3β-hydroxysteroid dehydrogenase<br>3β-HSD<br>Hsd3β<br>3βHSD1<br>3betaHSD	•The basic search strategy was sufficient.
	**SRD5A1**	SRD5A1<br>Alpha-Reductase<br>Steroid 5-Alpha-Reductase	•The basic search strategy was sufficient.
HORMONES	**P5**	pregnenolone<br>pregnenolone.<br>pregnenolone,<br>pregnenolone:<br>(pregnenolone)<br>Pregenenolone| Pregnenolone/	•Pregnenolone was mostly mentioned in the context of STAR function descriptions and introductions. Therefore, the abstracts of all relevant studies were checked manually, and only those that measured pregnenolone were counted.
	**P4**	progesterone<br>progesterone,<br>progesterone.<br>(progesterone)<br>progesterone:<br>Progestin<br>Progesterone| Progesterone/	•After reviewing the abstracts of 75 articles, only 4 did not evaluate progesterone levels, and in 15 of them, progesterone was listed in the MeSH terms. We decided to disregard these. The abstracts of studies focusing on stem-cell-based and advanced models for testicular steroidogenesis were all checked.
	**DHEA/ DHEA-S**	DHEA<br>dehydroepiandrosterone<br>DHEA-S<br>dehydroepiandrosterone sulfate<br>DHEA sulfate<br>androstenolone sulfate	•It would be complicated to distinguish between DHEA and DHEA-S in their full names; therefore, the two hormones were merged into one category.
	**A4**	Androstenedione<br>4-Androstene-3,17-dione<br>Androst-4-ene-3,17-dione<br>4-Androstenedione	•The basic search strategy was sufficient.
	**A5**	Androstenediol<br>5-Androstenediol<br>Androst-5-enediol	•The basic search strategy was sufficient.
	**T**	testosterone<br>Testosteron	•Testosterone frequently appears in introductions and as a MeSH term without being mentioned in the abstract. Additionally, it also captures dihydrotestosterone. Therefore, the abstracts of relevant studies were checked manually.

	**E1/E2/E3**	estrogen<br>estrone<br>estradiol<br>estriol	•Apart from measuring E1/E2/E3 levels as a response, these hormones are often used as substances of interest for which effects were detected. Additionally, estrogen is part of the "estrogen receptor." Therefore, the abstracts of relevant studies were checked manually.
**HORMONES**	**DHT**	DHT<br>5-α-dihydrotestosterone<br>Dihydrotestosterone<br>5α-DHT<br>stanolone<br>androstanolone	•The basic search strategy was sufficient.
	**17-OHP5**	17-OH-pregnenolone<br>17α-Hydroxypregnenolone<br>17-hydroxypregnenolone	•The basic search strategy was sufficient.
	**17-OHP4**	17-OH-progesterone<br>17α-Hydroxyprogesterone<br>17α-OHP<br>17-OH progesterone<br>hydroxyprogesterone<br>17-OHP	•The basic search strategy was sufficient.
	**ADIOL**	Androstanediol<br>3α-Androstanediol<br>5α-androstane-3α,17β-diol<br>3β-Androstanediol<br>5α-androstane-3β,17β-diol<br>3α-diol<br>5alpha-Androstane-3alpha, 17beta-diol<br>3alpha-DIOL	•The basic search strategy was sufficient.
	**AN**	Androsterone<br>3α-hydroxy-5α-androstan-17-one<br>Androsterone.<br>Androsterone,<br>Androsterone:<br>Androsterone| Androsterone/<br>(androsterone)	•The basic search strategy was sufficient.
	**DHP**	dihydroprogesterone<br>DHP<br>5α-Dihydroprogesterone<br>5α-DHP<br>allopregnanedione<br>5α-pregnane-3,20-dione	•The basic search strategy was sufficient.
	**5α-A**	Androstanedione<br>5α-androstanedione<br>5α-androstane-3,17-dione	•The basic search strategy was sufficient.
**CHOL transport**	**CHOL transport**	cholesterol<br>cholesterol.<br>cholesterol,<br>cholesterol:<br>(cholesterol)<br>cholesterol/<br>cholesterol| cholesterol	•Finding studies that evaluated cholesterol transport in general was challenging. The issue was further complicated by the presence of the "Cholesterol Side-Chain Cleavage Enzyme." Therefore, if the term "cholesterol" appeared in the abstract outside the context of the "Cholesterol Side-Chain Cleavage Enzyme," the study was included. This approach may lead to an overestimation of results due to studies that investigated cholesterol for other reasons. All abstracts of studies focusing on stem-cell-based and advanced models for testicular steroidogenesis were reviewed.
	**STAR**	STAR<br>STAR.<br>STAR,<br>STAR:	•The basic search strategy was sufficient.

		(STAR)<br>STAR/<br>STAR| Steroidogenic Acute Regulatory Protein<br>STARD1	
	**TSPO**	TSPO<br>Translocator Protein	•The basic search strategy was sufficient.
	**Lipid droplets**	lipid accumulation<br>lipid droplet	•The basic search strategy was sufficient.

Supplementary Table S2: Number of studies using *in vitro* models for testicular steroidogenesis identified through general keyword-based searches (Search Strategy 1), categorized by term of interest.

	STIMULATION					ENZYMES							CHOLESTEROL TRANSPORT			
	hCG	cAMP analog	Hydroxy CHOL	Forskolin	LH	CYP11A1	CYP19A1	AKR1C3	HSD17B3	CYP17A1	HSD3B	SRD5A1	CHOL transport	STAR	TSPO	Lipid droplets
**Primary**	136	64	19	13	5	56	48	10	1	17	63	10	69	2	12	2
**H295R**	7	0	13	0	47	26	94	92	5	30	58	2	49	2	19	3
**MA-10**	139	37	116	30	26	68	21	2	0	2	29	5	158	15	129	8
**TM3**	13	8	1	0	0	34	19	4	0	19	29	1	47	1	8	2
**MLTC1**	43	10	11	6	7	28	11	0	0	4	16	1	32	0	16	4
**R2C**	1	3	3	1	4	7	3	18	0	2	6	0	15	0	4	1
**LC-like**	3	6	10	4	5	12	8	0	0	6	13	0	5	0	0	1

	HORMONE													
	P5	P4	DHEA/ DHEA-S	A4	A5	T	E1/E2/E3	DHT	17-OHP5	17-OHP4	ADIOL	AN	DHP	5α-A
**Primary**	9	30	10	20	0	269	24	6	1	8	3	2	0	0
**H295R**	11	76	62	43	0	122	119	10	9	20	0	0	0	1
**MA-10**	17	180	1	5	0	30	3	5	0	2	0	0	3	0
**TM3**	0	7	2	1	0	117	5	0	0	0	0	0	0	0
**MLTC1**	3	46	1	4	0	29	0	1	0	2	0	0	0	0
**R2C**	2	14	0	1	0	11	5	0	0	0	0	0	0	0
**LC-like**	0	4	1	1	0	24	3	1	0	0	0	0	0	0

Supplementary Table S3: Number of studies using *in vitro* models for testicular steroidogenesis identified through targeted chemical searches (Search Strategy 2), categorized by term of interest and focused on chemical-specific effects on hormone levels during male steroidogenic processes.

	STIMULATION					ENZYMES							CHOLESTEROL TRANSPORT			
	hCG	LH	cAMP analog	Hydroxy CHOL	Forskolin	CYP11A1	CYP17A1	CYP19A1	AKR1C3/ 6/14	HSD17B	HSD3B	SRD5A1	STAR	TSPO	CHOL transport	Lipid droplets
**Primary**	38	39	20	11	26	25	16	2	3	10	17	7	31	3	2	1
**LC-like**	1	2	3	2	6	4	5	0	0	3	6	0	6	0	0	2
**H295R**	0	0	0	0	47	16	20	17	0	9	18	0	16	0	0	0
**MA-10**	12	7	13	4	10	14	5	1	1	3	10	2	13	2	1	2
**TM3**	4	0	0	0	0	5	5	0	1	3	7	0	6	0	0	1
**mLTC1**	8	1	1	0	1	1	2	1	0	0	0	0	3	0	0	0
**BLTK1**	3	1	1	0	1	2	2	2	0	2	2	2	2	0	0	0

	HORMONE													
	P5	P4	DHEA/ DHEA-S	A4	A5	T	E1/E2/E3	DHT	17-OHP5	17-OHP4	ADIOL	AN	DHP	5α-A
**Primary**	8	9	1	2	0	91	6	5	1	0	4	1	0	3
**LC-like**	1	4	0	1	0	6	0	1	0	0	0	1	0	0
**H295R**	20	40	24	29	2	67	61	6	14	18	1	2	1	1
**MA-10**	1	26	0	1	0	8	3	1	0	0	0	0	0	0
**TM3**	0	0	0	0	0	17	0	0	0	0	0	0	0	0
**mLTC1**	0	5	0	2	0	6	0	0	0	2	0	0	0	0
**BLTK1**	0	3	0	0	0	3	1	0	0	0	0	0	0	0

SUPPLEMENTARY FIGURES

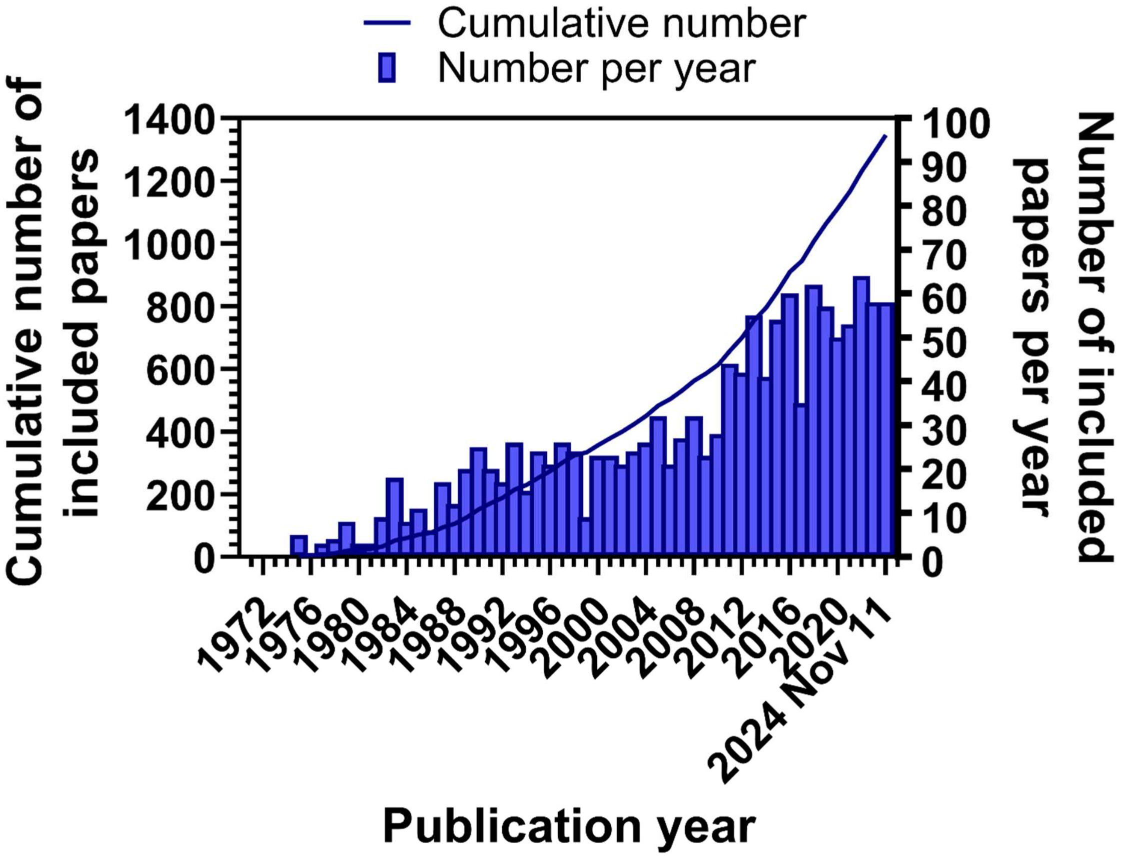

Supplementary Figure S1: **Distribution of publication years for all studies included in the review, based on general keyword-based searches of *in vitro* models for testicular steroidogenesis.**

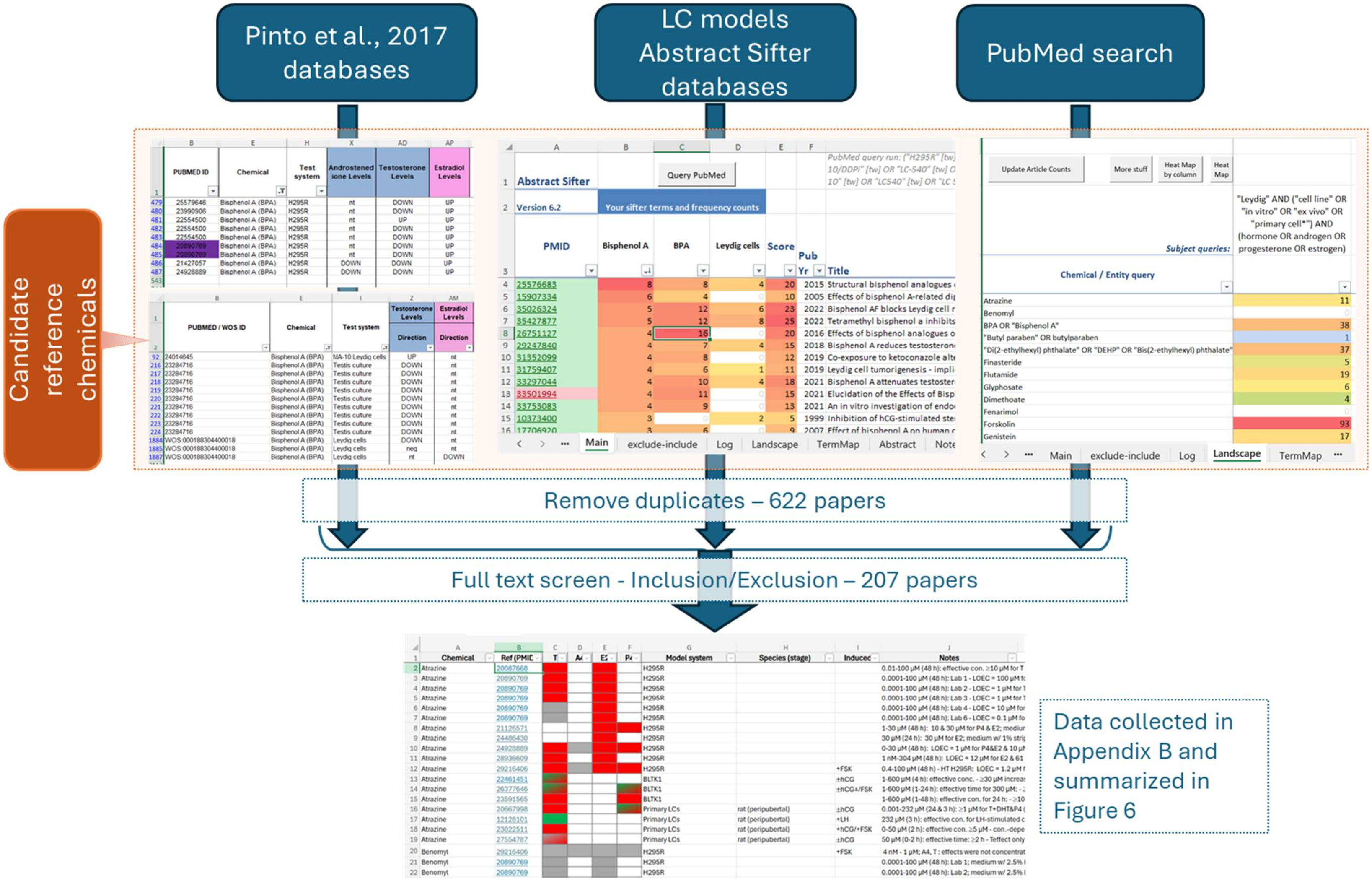

Supplementary Figure S2: **Flowchart illustrating the study selection process for targeted searches (Search Strategy 2), focusing on chemical-specific effects on hormone levels in *in vitro* models of male steroidogenesis.**

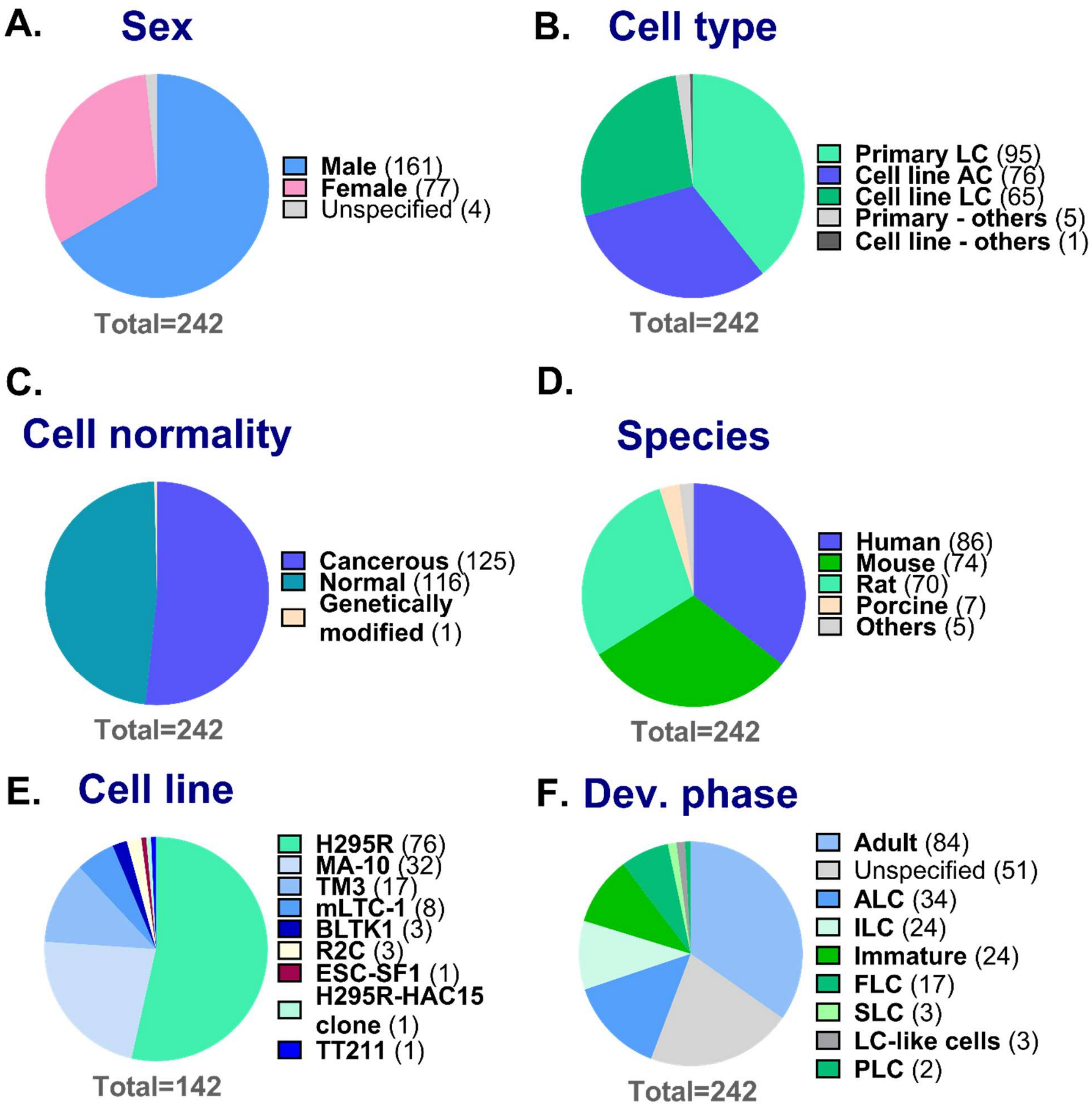

Supplementary Figure S3: **Characteristics of *in vitro* models used to investigate the effects of selected chemicals on hormone levels during male steroidogenesis, identified through targeted searches (Search strategy 2). AC**, adrenal cell; **F**, female; **LC**, Leydig cells; **M**, male.

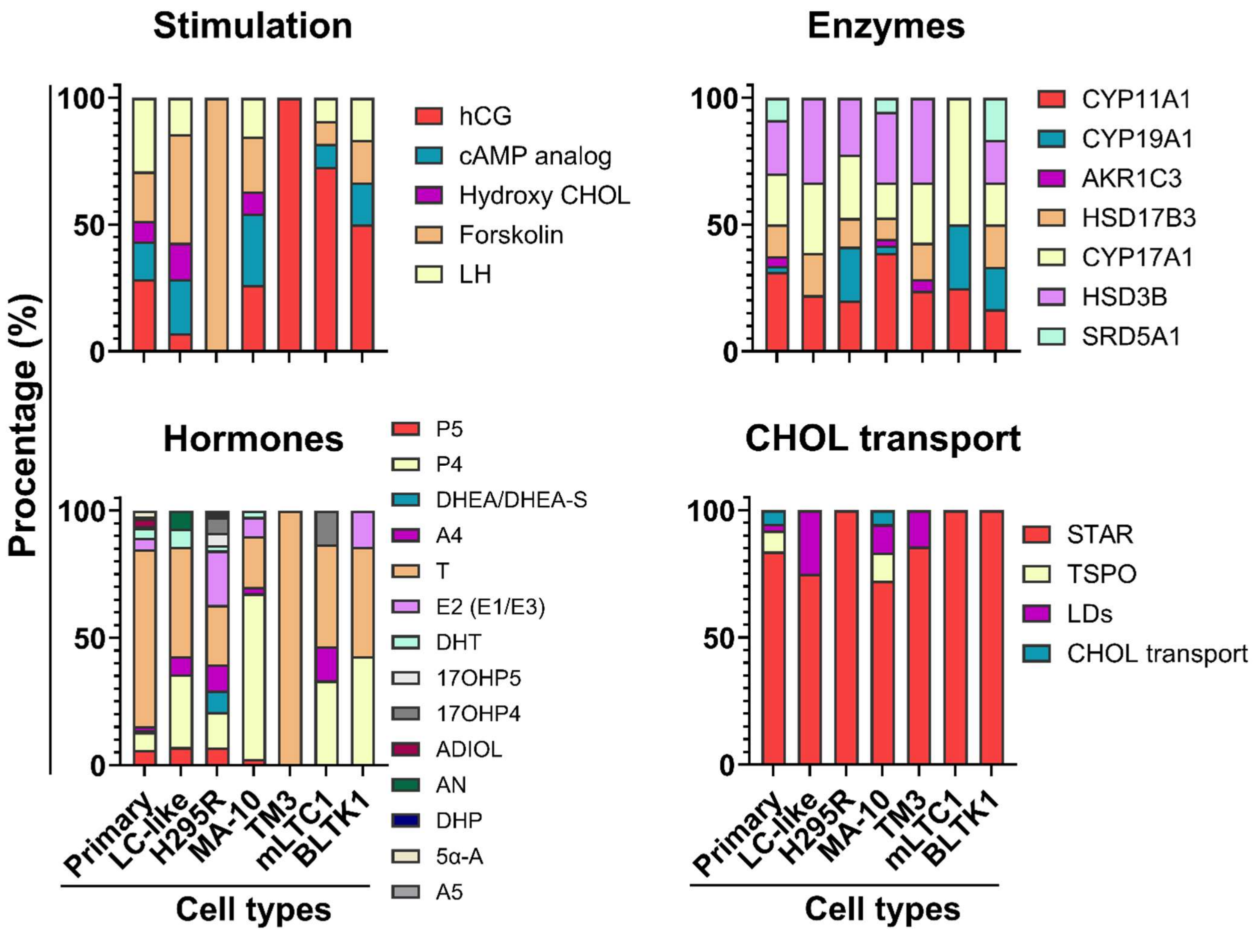

Supplementary Figure S4: Characteristics of *in vitro* models investigating the effects of selected chemicals on hormone levels during male steroidogenesis, identified through targeted searches (Search strategy 2). The figure summarized agents used to stimulate steroidogenesis, steroidogenic enzymes and hormones evaluated, and parameters assessed for cholesterol transport. The number of studies addressing each parameter is provided in **Supplementary Table S3.**

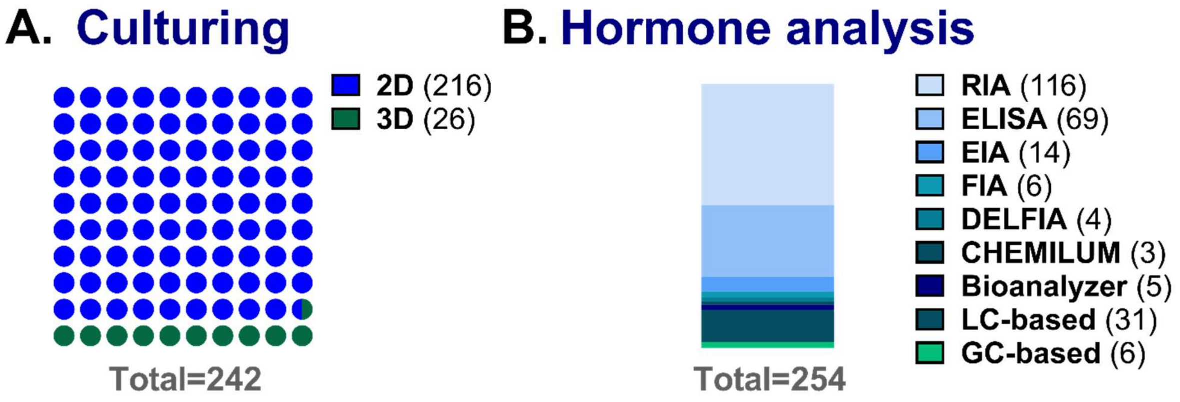

Supplementary Figure S5: Culture conditions of *in vitro* models (A.) investigating the effects of selected chemicals on hormone levels during male steroidogenesis, identified through targeted searches (Search strategy 2), and methods used for hormone analysis (B). 2D, two-dimensional; **3D**, three-dimensional; **CHEMILUM**, chemiluminescence immunoassay; **DELFIA**, dissociation-enhanced lanthanide fluorescent immunoassay; **EIA**, enzyme immunoassay; **ELISA**, enzyme-linked immunosorbent assay; **FIA**, fluorescence immunoassay; **GC**, gas chromatography; **LC**, liquid chromatography; **RIA**, radioimmunoassay.
